# Recent progress in carbon dots for anti-pathogen applications in oral cavity

**DOI:** 10.3389/fcimb.2023.1251309

**Published:** 2023-09-15

**Authors:** Yuying Jiang, Chuqiang Yin, Jianning Mo, Xiaoyu Wang, Ting Wang, Guotai Li, Qihui Zhou

**Affiliations:** ^1^ The Affiliated Hospital of Qingdao University, Qingdao University, Qingdao, China; ^2^ School of Stomatology, Qingdao University, Qingdao, China; ^3^ School of Rehabilitation Sciences and Engineering, University of Health and Rehabilitation Sciences, Qingdao, China

**Keywords:** nanomedicine, carbon dots, antimicrobial, bacterial imaging, oral microbial infectious

## Abstract

**Background:**

Oral microbial infections are one of the most common diseases. Their progress not only results in the irreversible destruction of teeth and other oral tissues but also closely links to oral cancers and systemic diseases. However, traditional treatment against oral infections by antibiotics is not effective enough due to microbial resistance and drug blocking by oral biofilms, along with the passive dilution of the drug on the infection site in the oral environment.

**Aim of review:**

Besides the traditional antibiotic treatment, carbon dots (CDs) recently became an emerging antimicrobial and microbial imaging agent because of their excellent (bio)physicochemical performance. Their application in treating oral infections has received widespread attention, as witnessed by increasing publication in this field. However, to date, there is no comprehensive review available yet to analyze their effectiveness and mechanism. Herein, as a step toward addressing the present gap, this review aims to discuss the recent advances in CDs against diverse oral pathogens and thus propose novel strategies in the treatment of oral microbial infections.

**Key scientific concepts of review:**

In this manuscript, the recent progress of CDs against oral pathogens is summarized for the first time. We highlighted the antimicrobial abilities of CDs in terms of oral planktonic bacteria, intracellular bacteria, oral pathogenic biofilms, and fungi. Next, we introduced their microbial imaging and detection capabilities and proposed the prospects of CDs in early diagnosis of oral infection and pathogen microbiological examination. Lastly, we discussed the perspectives on clinical transformation and the current limitations of CDs in the treatment of oral microbial infections.

## Graphical Abstract

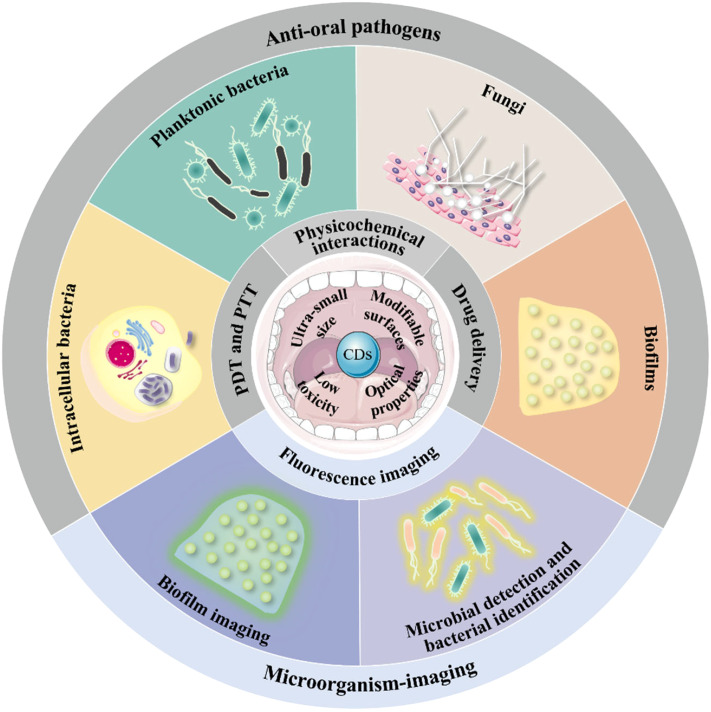

## Introduction

Multiple microorganisms inhabit the oral cavity, forming an ecological community, which is closely related to oral health and disease, usually known as the oral microbiome (Dewhirst et al., 2010). Generally, the flora and the host maintain a homeostatic balance (Kamada et al., 2013; Samaranayake and Matsubara, 2017), however, risk factors, such as host behavior and immune response alterations, could lead to dysbiosis of the oral microbiome (Lamont et al., 2018; Rao et al., 2022), manifested as the overproliferation of disease-associated microbiota, the formation of pathogenic biofilms, the penetration of bacteria within oral epithelial tissues, and even the invasion of intracellular pathogens (Yamatake et al., 2007; Kolenbrander et al., 2010; Takeuchi et al., 2011). Those pathological alterations can further change the oral microenvironment, eventually resulting in infectious diseases (Lamont et al., 2018).

Clinically, oral infectious diseases mainly include dental caries, periodontitis, peri-implantitis, endodontic infections, orthodontic infections, oral candidiasis, etc. (Allaker and Memarzadeh, 2014; Liang et al., 2020b). If not treated promptly and correctly, such microbe infections will progress gradually, leading to irreversible destruction of teeth or other oral tissues (Listgarten, 1986; Kong et al., 2015; Veiga et al., 2016). Besides, oral infectious diseases can lead to oral cancers, possibly owing to the interference of an altered immunoinflammatory state to the host (Whitmore and Lamont, 2014). There is also increasing pieces of evidence that support the extensive influence of the oral microbiome on systemic diseases, such as coronary artery disease, rheumatoid arthritis, and digestive system neoplasms (Högberg et al., 2010; Hu et al., 2019; Jain et al., 2021). Therefore, prevention and effective treatment of oral pathogen infectious are of great clinical significance.

Nowadays, clinical treatment for oral pathogen infectious disease includes mechanical plaque control procedures and the administration of antibiotics (Sah et al., 2019). As a valuable complement to the former, systematic and localized antimicrobial approaches could help eliminate pathogenic bacteria and limit the translocation of pathogens to different sites in the oral cavity (Slots and Rams, 1990). However, the effect is usually not ideal due to the following reasons. Firstly, orally administered antibiotics have diluted a thousandfold at the site of infection, which results in low efficiency (Aithal et al., 2018; Yang et al., 2018; Sah et al., 2019). Secondly, the recalcitrant infections caused by antibiotic resistance in bacteria increased the difficulty of thorough treatment (Makabenta et al., 2021; Li et al., 2022a; Shao et al., 2022). Numerous studies have confirmed that microbial antibiotic resistance is closely related to the lack of drug efficacy (Stokes et al., 2019; Ré et al., 2021). As known to all, most oral infections are caused by diverse microbial species (Haffajee and Socransky, 1994; Ding et al., 2022). The pathogens are embedded in a self-produced matrix of extracellular polymeric substances (EPS) and form multi-species plaque biofilms, which increase antibiotic resistance to 2 to 1000-fold higher than planktonic bacteria (Pantlin, 2008; Flemming et al., 2016; Hu et al., 2019). As for localized antibiotics, the main factors that lead to the reduction of the efficient concentration of drugs are the protective barrier against antibiotics provided by biofilms and the passive loss of drugs due to the fluid environment of the oral cavity (Meisel and Kocher, 2005; Allaker and Memarzadeh, 2014). Moreover, some oral pathogens like *Porphyromonas gingivalis* (*P. gingivalis*) and *Candida albicans* (*C. albicans*) can invade and localize into the oral epithelial cells, leading to the recurrence of the disease (Fidel, 2011; Wayakanon et al., 2013). However, the limited diffusion, bad endocytosis ability of mammalian cell membrane to antibiotics and the short intracellular retention time of antibiotics lead to a reduction of the intracellular level of the drug, adding significant complexity to treatment (Kamaruzzaman et al., 2017).

Furthermore, long-term administration of antibiotics may cause side effects including gastrointestinal disturbances (Meisel and Kocher, 2005), allergies (Slots and Rams, 1990), and tooth discoloration (Parhizkar et al., 2018).

Disinfectants and antiseptics such as sodium hypochlorite (NaClO) and chlorhexidine (CHX) are also commonly used to eliminate oral biofilms in clinical treatment (Tosco et al., 2023). For instance, NaClO is frequently applied to flush the root canal in the root canal treatment (RCT) procedure, aiming to kill the residual bacteria in narrow areas such as dentinal tubules (Gawdat and Bedier, 2022). However, such disinfectants are facing restrictions because of their innate bio-physiochemical features (Ji et al., 2021). For example, NaOCl has an adverse effect on tissue irritation, CHX could cause dry mouth or taste changes when used as a mouthwash, and calcium hydroxide takes more than seven days to exhibit an antibacterial effect (Siqueira and De Uzeda, 1996; Siqueira and Lopes, 1999; Sundaram et al., 2016; Scheibler et al., 2017). Therefore, novel antibacterial agents with high efficacy in the oral infection site and good biocompatibility are urgently needed.

Nanomedicine has gradually become an alternative therapeutic approach to traditional diagnosis and treatment in the last decades (Zhou et al., 2016; Almonacid Suarez et al., 2019; Liguori et al., 2019; Liu et al., 2021a; Song et al., 2022). Nanomaterials with large surface area, surface functionalization capacity, and low therapeutic doses have outstanding advantages in antimicrobial applications (Zhu et al., 2021; Mei et al., 2022; Yan et al., 2022; Jiang et al., 2023; Wu et al., 2023). Particularly, due to their excellent biocompatibility and unique physicochemical properties, carbon dots (CDs) are gaining importance in the field of biomedicine (Su et al., 2020). CDs are zero-dimensional carbon nanomaterials with sizes ranging from 2-10 nm (Baker and Baker, 2010). Based on their intrinsic inner structure and surface chemical groups, they are usually divided into three categories: graphene quantum dots (GQDs), carbon nanodots (CNDs), and polymer dots (PDs) (Zhu et al., 2015). Since the discovery of CDs in 2004, CDs have received increasing attention in the antibacterial field for their low toxicity, modifiable surfaces, and excellent optical properties (Xu et al., 2004; Feng and Qian, 2018). Previous antibacterial studies have suggested that the properties of CDs endow the material with great possibilities to address the shortcomings of oral antibiotics.

According to the above summary of the drawbacks of oral antibiotics, it can be inferred that the high-efficacy oral antibacterial agents need to meet requirements as the combination of the long residence time or rapid sterilization ability and the solid permeability for ensuring a sufficient dose of drug reaching infection sites. Numerous studies have confirmed that CDs meet the above conditions and can be an oral antibacterial agent with great potential. Researchers have reported that CDs could exhibit efficient broad-spectrum or obligate antibacterial properties, depending on their precursor or preparation process. For example, Li et al. synthesized CDs from vitamin C via the electrochemical method and tested their antimicrobial abilities against various strains. Results indicated that the CDs could efficiently inhibit the growth of both Gram-positive and Gram-negative bacteria (Li et al., 2018a). Sun et al. used the biguanide antibacterial drug (chlorhexidine gluconate) as a raw material to prepare CDs (CGCDs), and the prepared CGCDs could reach a 100% antibacterial rate at 100 µg/mL (Sun et al., 2021). While CDs derived from glycerol and dimethyloctadecyl[3-(trimethoxysilyl)propy]ammonium chloride (Si-QAC) by the solvothermal treatment could selectively kill Gram-positive bacteria (Yang et al., 2019).

Moreover, owing to their intricate antibacterial mechanisms (i.e. damage of the bacterial membranes (Li et al., 2020a), reactive oxygen species (ROS) generation (Romero et al., 2021), disruption of protein synthesis (Zhao et al., 2022a), etc.), different from antibiotics, CDs could not only achieve rapid sterilization in minutes but are also effective against drug-resistant bacteria, even including multidrug-resistant bacteria (MDR) (Li et al., 2016; Sidhu et al., 2017; Yang et al., 2021; Zhao et al., 2022c).

Furthermore, studies have shown that CDs have good permeability and can effectively remove oral biofilms (Pourhajibagher et al., 2019; Liang et al., 2020a). The complex root canal system in the oral cavity is difficult to penetrate, and the penetration depth in the dentinal tubules is one of the important characteristics of root canal disinfectants. With extremely small particle size, CDs has great advantages in going deep into the antibacterial narrow areas. CDs derived from fucoidan (FD) were synthesized by Tang et al. The FD-CDs can open dentin tubules and significantly remove *E. faecalis* from root canals and dentin tubules, which is equivalent to the antibacterial effect of NaClO (Tang et al., 2022). Through the above example, it can be seen that CDs have great advantages in removing oral biofilms.

CDs also have an excellent effect against intracellular bacteria and fungi (Ardekani et al., 2019; Li et al., 2019). They have been widely studied in the microbial imaging field due to their optical characteristics (Zhu et al., 2015; Lin et al., 2019). Researchers have applied CDs as imaging agents and detection indicators for microorganisms and biofilms to evaluate the antibacterial effects of materials and auxiliary means for early diagnosis (Ghirardello et al., 2021). In the current research ideas, many researchers have also made assumptions and prospects for the application of microbial imaging ability of carbon dots in the oral cavity (Li et al., 2019; Tang et al., 2022). Combining the luminescent and bactericidal properties of CDs can make them nanomaterials that integrate detection and antibacterial applications. Their potential in medical diagnosis and treatment is rapidly gaining prominence.

**Figure 1:**
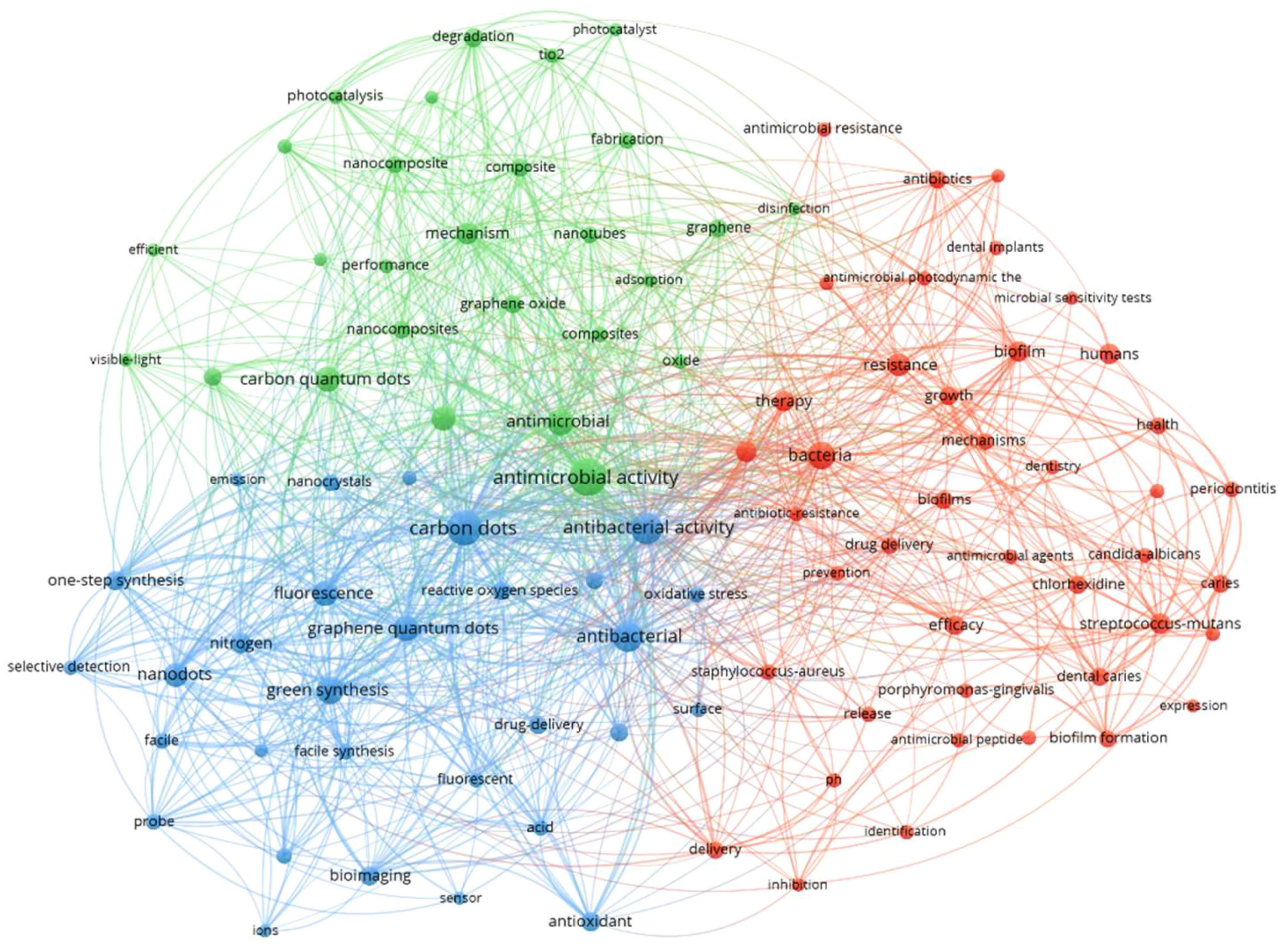
The analysis of keywords co-occurrences on carbon dots, antimicrobial, bacterial imaging, and oral microbial infectious.

In recent years, increasing efforts have been put into the research of CDs for various applications, as shown in **Figure 1**, drawn for the co-occurrences on CDs, antimicrobial, bacterial imaging, and oral microbial infectious analyzed by the VOS viewer bibliometric visualization software. Circles in different colors represent the keywords related to the four topics (CDs, antimicrobial, bacterial imaging, and oral microbial infectious), and the links between circles demonstrate their relations. The result indicates huge intersections between CDs and the field of anti-pathogens in the oral cavity, presenting great research potential.

Herein, this review illustrates the recent progress in CDs used for anti-pathogens in the oral cavity. We mainly focus on the applications of CDs in killing (**Table 1**) and imaging oral microorganisms. The perspectives on clinical transformation and current challenges are discussed in the last section.

**Table 1:** Summary of carbon dots for combating oral microbial infections.

Oral disease/Problem	Precursors	Microorganisms	Antimicrobial mechanisms	Therapeutic effects	References
Periodontal diseases	Tinidazole; metronidazole	*P. gingivalis*	Retention of the antibacterial nitro group; downregulation of the biofilm genes expression pattern	Specific antibacterial and antibiofilm activity; inhibition of biofilm formation	(Liang et al., 2020a)
Periodontitis	Metronidazole	*P. gingivalis*	Retention of the antibacterial nitro group	Selective antibacterial activity	(Liu et al., 2017a)
Periodontal diseases	Graphene oxide	*P. gingivalis, A. actinomycetemcomitans, P. intermedia*	PDT; downregulation of the biofilm genes expression pattern	Antibacterial activity against the mixed perio-pathogens; degradation and reduction of the progression of perio-pathogens mixed biofilms	(Pourhajibagher et al., 2019)
Intracellular periodontal pathogen invasion	Chlorophyll	*P. gingivalis*	Intracellular delivery of metronidazole	Increased inhibition of intracellular *P. gingivalis* at lower antibiotic dosages	(Ardekani et al., 2019)
Dental biofilms	Chlorhexidine and tributylhexadecylphosphonium bromide	*S. mutans*	Electrostatic interactions	Antibacterial activity; disruption of mature biofilms	(Ostadhossein et al., 2021)
Persistent endodontic infections	Fucoidan	*E. faecalis*	Retention of the antibacterial sulfate radicals; destruction of bacterial walls; ROS generation; recruitment of the macrophages	Antibacterial activity; antibiofilm activity	(Tang et al., 2022)
Oral infectious diseases	Guanidine hydrochloride, citric acid, and copper chloride	*E. coli, S. aureus, S. mutans*	ROS and O_2_ generation	Antibacterial and antibiofilm activity; inhibition of biofilm formation	(Liu et al., 2022)
Dental pathogenic microflora	Acetone leaf extract of *Cannabis sativa*	*E. coli, S. aureus, dental micro flora*	Retention of the antibacterial activity of precursors; modification of Ag nanoparticles	Antibacterial activity	(Raina et al., 2020)
Bacteria-caused enamel demineralization of the orthodontic brackets	Citric acid and urea	*S. mutans*	PDT	Antibacterial activity	(Taufiq et al., 2020)
Bacteria-caused enamel demineralization of the orthodontic brackets	Sucrose	*E. coli, S. aureus, S. mutans*	PDT	Antibacterial activity	(Zhang et al., 2018)
Implant-related infections	Graphite	*E. coli, S. aureus*	PDT	Antibacterial activity	(Moradlou et al., 2019)
Persistent microbial contamination of medical implant surfaces	Polyoxyethylene-polyoxypropylene-polyoxyethylene Pluronic 68	*E. coli, S. aureus*	PDT	Antibacterial activity	(Budimir et al., 2021)
Implant-related infections	L-tryptophan and chlorhexidine acetate	*S. aureus*	PDT, PTT	Antibacterial activity	(He et al., 2022)
Implant-related infections	Citric acid monohydrate and glycine	*E. coli, S. aureus*	PDT, PTT	Antibacterial activity	(Han et al., 2020)
Oral candidiasis	Citric acid, ethylenediamine, and formamide	*C. albicans*	Delivery of amphotericin B	Antifungal activity, prevention of the adherence and invasion of *C. albicans* in oral epithelia	(Li et al., 2019)
Attack of hydrophobic microorganisms on medical implants	Fruit juice extracted from *Citrus limetta*	*C. albicans*	Electrostatic interactions	Inhibition of the attachment and formation of biofilms	(Shaikh et al., 2018)
Fungal infections	3-iodo-L-tyrosine, 3,5-diiodo-DL-tyrosine, iopromide, and ethylenediamine	*C. albicans*	ROS generation contributing to peroxidase-like and photocatalytic activities of iodine-doped CDs	Antifungal activity	(Li et al., 2021b)
Fungal infections	Citric acid and PEG	*C. albicans*	Small particle size, modification of Au nanoparticles	Antifungal activity	(Priyadarshini et al., 2018)

## Antimicrobial applications of CDs

Oral infectious diseases are mainly caused by a diverse pathogenic oral microbes. Such pathogenic microbes tend to form supra- and subgingival plaque biofilms on different surfaces in the oral cavity (Kolenbrander et al., 2010). For example, the most common supragingival plaque-related disease is dental caries, a destructive disease of the dental hard tissue. Its progression can lead to infection of the pulp tissue and even the periapical area (Larsen and Fiehn, 2017). Subgingival plaque biofilms are commonly related to periodontitis (Takeuchi et al., 2011). It can lead to the destruction of the soft and hard tissues in the oral cavity, such as the periodontal ligament and alveolar bone (Preshaw et al., 2005). The main pathogenic bacteria of periodontitis can invade and survive in host cells intracellularly, causing recurrent inflammation (Makkawi et al., 2017). As for immunocompromised patients, oral candidiasis is quite common (Talianu et al., 2022).

Herein, we divided the antimicrobial applications of CDs against oral microorganisms into four categories: anti-oral planktonic bacteria, anti-intracellular bacteria, antibiofilm, and antifungal. Antibacterial mechanisms of CDs are vital in instructing the treatment of oral infectious diseases. Thus, the mechanisms of CDs interacting with oral pathogens are also summarized in this section.

### CDs against oral planktonic bacteria

Numerous studies have verified that certain oral pathogens are associated with specific oral infections (O’Brien-Simpson et al., 2004; Xia et al., 2019). For example, the abnormal proliferation of *Streptococcus mutans* (*S. mutans*) can cause caries (Krzyściak et al., 2014), while in resistant endodontic infections, *Enterococcus faecalis* (*E. faecalis*) is considered to be the primary pathogen causing persistent or secondary endodontic infection (Fouad et al., 2005; Persoon et al., 2017). Besides, *C. albicans* can form mixed biofilms in the root canal, further aggravating the infection (Ji et al., 2021). As for subgingival microbe-related diseases, periodontitis is widely known to be caused by anaerobic bacteria dominated by *P. gingivalis* (Popova et al., 2013). Whereas for peri-implantitis and denture-induced stomatitis, major pathogens include *Staphylococcus aureus* (*S. aureus*) and other Gram-negative bacteria (Smith et al., 2001; Armitage et al., 2010; Persson and Renvert, 2014).

CDs are potential agents for combating oral infections. The design of antibacterial CDs with diverse mechanisms at the macroscopic and molecular levels has always been a hot research topic. However, the actual mechanisms are still an open debate among researchers. It is commonly agreed that CDs can possess bactericidal ability through multiple approaches, including physicochemical interactions with cells (hydrophobic interactions, electrostatic attractions, surface functional groups, etc.) and the formation of ROS. ROS can cause damage to the cell membrane and bacteria lysis, interfering with gene expression and disturbing biological processes (Ran et al., 2019; Li et al., 2020a; Tang et al., 2022). Besides their intrinsic bactericidal features, CDs can also participate in phototherapy. With their unique optical properties, CDs could serve as photosensitizers (PSs) or photothermal agents (PTAs) to generate ROS or heat under light irradiation, and those without inherent photoactive abilities could be modified on PSs to enhance their photodynamic efficiency (Sattarahmady et al., 2017; Moradlou et al., 2019; Nie et al., 2020). Their great biocompatibility and abundant surface chemical group enable them to work as a nanocarrier to deliver antibiotics and other therapeutic agents as well (Thakur et al., 2014; Jijie et al., 2018; Ardekani et al., 2019).

**Figure 2:**
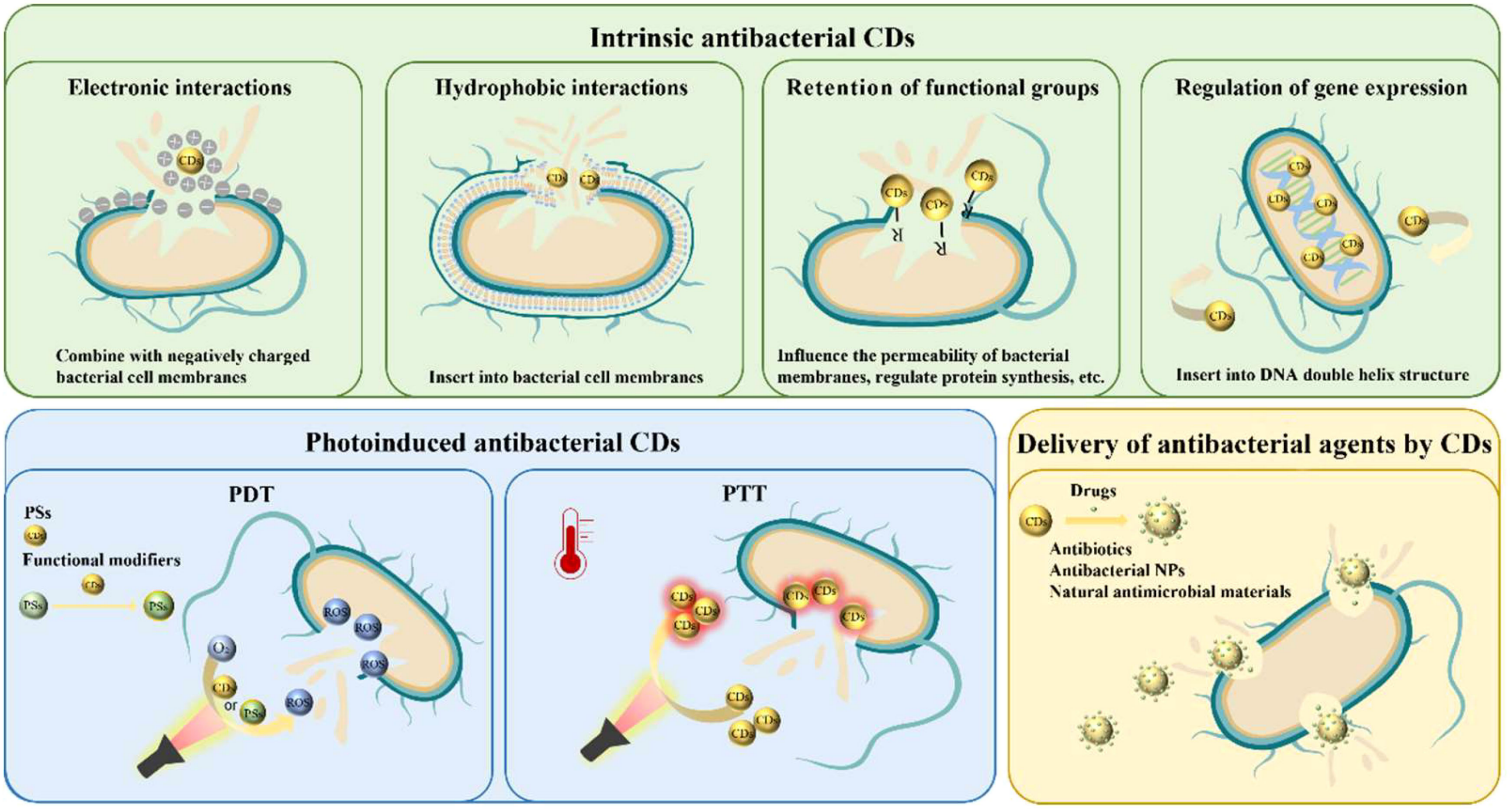
Scheme of CDs against oral planktonic bacteria.

Therefore, we discuss the antibacterial CDs against oral planktonic bacteria in this section into three categories according to their antibacterial mechanisms: 1) Intrinsic antibacterial CDs. 2) Photoinduced antibacterial CDs. 3) CDs for delivery and modification of antimicrobial agents (**Figure 2**).

### Intrinsic antibacterial CDs

Specific surface chemistry characteristics, such as charge, hydrophobicity, and functional groups, have been reported to be closely related to the bactericidal features of CDs; these characteristics enable them to contact the bacterial surface directly and mechanically rupture the wall and membrane (Cui et al., 2020). Furthermore, CDs could also lead to ROS generation, which can consequently induce apoptotic cell death through the following downstream effects such as degradation of genomic DNA, protein fragmentation, and chromosome condensation (Bing et al., 2016; Travlou et al., 2018).

Surface charge is a factor that is often considered while designing antibacterial CDs (Havrdova et al., 2016; Li et al., 2020b). It is well-demonstrated that bacterial membranes are more negatively charged than mammalian cell membranes (Matsuzaki, 2009). Therefore, the introduction of positively charged components, such as amino group, quaternary ammonium compound, and phosphorus element, can endow CDs with positive surface charge, improving the interaction between CDs and bacterial membranes, hence reaching a bactericidal purpose (Yang et al., 2019; Chai et al., 2021; Yang et al., 2021).

**Figure 3:**
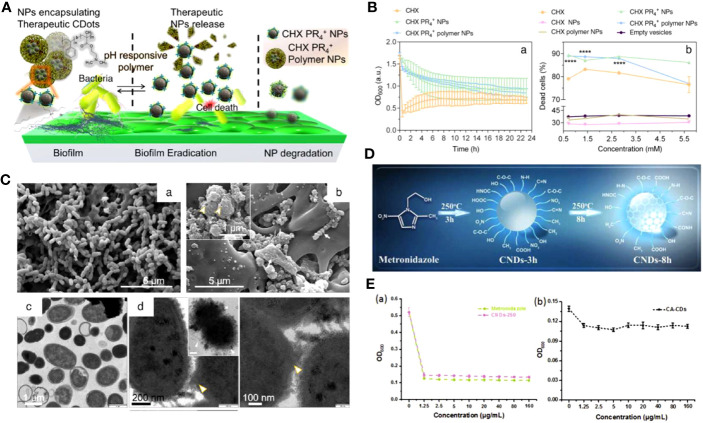
**(A)** Schematic representation of the action of CHX PR_4_+ polymer NPs as antibiofilm agents. **(B)** The efficacy of NPs against the planktonic form of *S. mutans*. (a) Turbidity time-kill assay showing a decreasing trend. (b) The quantitative live-dead fluorescence assay for various formulations. **(C)** The antibacterial mechanism governing cell death. SEM images of planktonic *S. mutans* treated with different groups (Ostadhossein et al., 2021). Open access article with no copyright permission. **(D)** Illustration of the possible formation mechanism of CNDs. **(E)** The growth curves of *P. gingivalis* after incubation with various concentrations of Metronidazole and CNDs-250 (a) CA-CDs (b) for 24 h, respectively (Liu et al., 2017a). Copyright RCS reprinted with permission. Order License ID: 1347745-1.

In recent years, researchers reported that cationic CDs interacted with various oral pathogens. As a major pathogenic bacteria (*S. mutans*) of dental caries, its hyperproliferation could not only cause the production of organic acids that leads to tooth decalcification but also disturb the balance of oral microbiota (Hu et al., 2019; Feldman et al., 2020; Afrasiabi et al., 2021). Li et al. reported the synthesis of cationic CDs through the hydrothermal method with cationic phenyl-bis biguanide chlorhexidine (CHX) as the CDs preparation source and tributyl hexadecyl phosphonium bromide (PR_4_+) to incorporate phosphonium functionality (Ostadhossein et al., 2021). They also prepared the CHX PR_4_+ NPs with a pH-responsive layer of a negatively charged amphiphilic polymer to further eradicate mature biofilms of *S. mutans* (**Figure 3A**). The electrophoretic ζ-potential values for the CHX PR_4_+ polymer NPs were +35 ± 4 mV. Both CHX PR_4_+ NPs and CHX PR_4_+ polymer NPs exhibited higher antibacterial activity than nanoparticles without PR_4_+ incorporation (**Figure 3B**). Further mechanistic studies confirmed the electrostatic interactions between the negatively charged bacterial membrane and the cationic NPs, resulting in the deformation of the bacteria, leakage of the intracellular content, and subcellular downstream effects (**Figure 3C**).

However, it has been reported that cationic CDs could enter the nucleus and induce cytotoxicity (Havrdova et al., 2016). To synthesize CDs with better biocompatibility, Xu et al. reported bioinspired antimicrobial CNDs (BAM dot) with neutral surface charge derived from a biomolecule precursor (Xu et al., 2018). It is worth mentioning that the BAM dot has amphiphilic and zwitterionic-like characteristics, which is crucial for its antibacterial applications. As a result, the BAM dot exhibited improved biosafety and excellent antibacterial activity, exemplified by the reduced interaction with the fibroblast cell membrane and the minimum inhibitory concentration (MIC) value of 250 μg mL^-1^ for *E. faecalis*. It was hypothesized that the BAM dot could first contact with the bacterial wall by noncovalent interactions (e.g., electrostatic, hydrogen bonding, van der Waals attraction), then insert into the cell membrane through hydrophobic interaction, which subsequently leads to cytoplasm leakage.

Retaining some characteristic functional groups of the precursors can also endow CDs with excellent antibacterial properties (Liang et al., 2020a; Tang et al., 2022). Metronidazole, an antibiotic specifically against anaerobes, was prepared as CNDs (CNDs-250) for the potential treatment of periodontitis (**Figure 3D**) (Liu et al., 2017a). The pathogenic bacteria of periodontal diseases are usually anaerobic bacteria such as *P. gingivalis*, *Aggregatibacter actinomycetemcomitans* (*A. actinomycetemcomitans*), and *Prevotella intermedia* (*P. intermedia*) (Popova et al., 2013). They can reduce the nitro group of metronidazole to nitroso free radical by transferring proteins with a low redox potential, leading to DNA degradation and the inhibition of DNA synthesis (Löfmark et al., 2010). The retention of the nitro group in CNDs-250 was verified by FTIR and XPS, and the CNDs selectively repress 71.7% of *P. gingivalis* growth at the concentration of 1.25 μg mL^-1^. Interestingly, comparing the antibacterial growth curve with CA-CD that was also negatively charged but without nitro groups, the survival rate of *P. gingvivalis* treated with CA-CD was more than 90%, indicating that the nitro group was the critical factor of inhibition, instead of surface charge (**Figure 3E**).

Up to now, most discussions about the bactericidal mechanisms of CDs are mainly focused on their physicochemical properties rather than the in-depth study of its antibacterial targets at the molecular level. Zhao et al. synthesized quaternized carbon quantum dots (qCQDs) through a simple "one-pot" method, using dimethyl diallyl ammonium chloride (DDA) and glucose as precursors (Zhao et al., 2022a). The qCQDs exhibited a broad-spectrum antibacterial effect on common pathogenic bacteria, including *E. faecalis*, *S. aureus*, and *Escherichia coli* (*E. coli*). Proteomic analyses and real-time quantitative PCR indicated that qCQDs mainly influenced the ribosomal proteins of Gram-positive bacteria to disrupt protein synthesis and down-regulated metabolized-related proteins for Gram-negative bacteria to interfere with cellular respiration. The results suggest that the molecular targets of qCQDs vary from traditional quaternary ammonium compounds (known to act on the cytoplasmic membrane) and other antibacterial CDs.

### Photoinduced antibacterial CDs

Phototherapy, including photodynamic therapy (PDT) and photothermal therapy (PTT), has received extensive attention in the antibacterial field, owing to its low toxicity and no drug-resistant characteristics (Wei et al., 2020). However, ideal PSs and PTAs are still under exploration due to the hydrophobic feature of traditional ones with poor water solubility. Nowadays, CDs have become a potential candidate for their excellent optical properties, such as broad absorption spectrum and photostability, and their excellent water solubility and biocompatibility (Li et al., 2021a).

### CDs in PDT

Antibacterial photodynamic therapy (aPDT) is based on light absorption of a specific wavelength with a non-toxic PS, which produces antimicrobial ROS with the presence of oxygen, resulting in damage of bacterial cell components and cell death (Huang et al., 2012; Mai et al., 2016; Romero et al., 2021; Zhao et al., 2023). Recently, aPDT has attracted tremendous interest in treating oral infectious diseases, such as dental caries (Melo et al., 2015), endodontic root canal infections (Tennert et al., 2014), oral candidiasis (Karem Janeth Rimachi Hidalgo et al., 2019), periodontitis, and peri-implantitis (Zhao et al., 2021).

**Figure 4:**
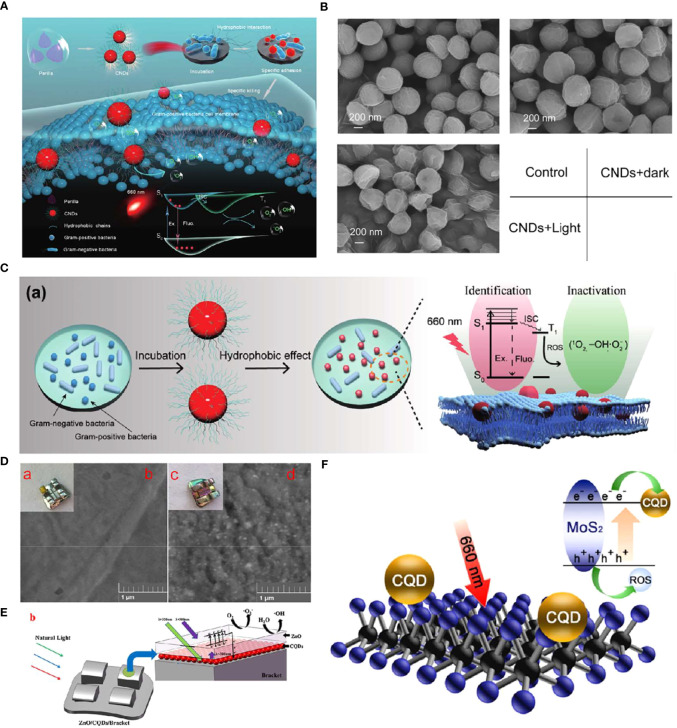
**(A)** Schematic diagram of the specific identification and killing of bacteria by the CNDs and the corresponding antibacterial mechanism. **(B)** SEM images of *S. aureus* treated with or without the CNDs under 660 nm light irradiation (Zhao et al., 2022c). Copyright Elsevier reprinted with permission. License Number: 5534630511657. **(C)** (a) Specific identification of Gram-positive bacteria by the CNDs and the corresponding photodynamic anti-bacteria mechanism. **(D)** The photograph (a, c) and SEM image (b, d) of the bracket (a, b) and bracket modified with ZnO/CQDs coating (c, d). **(E)** Scheme illustrating the enhanced photocatalytic property of Ti-PEI-MoS2/CQDs. **(F)** Illustration for upconversion, electronic transport, and photocatalytic antibacterial performance of brackets modified with ZnO/CQDs coating under natural light (Zhang et al., 2018). Copyright Elsevier reprinted with permission. License Number: 5534641452821.

CDs can be used as PSs for antibacterial effects. For instance, Zhao et al. synthesized perilla-derived CNDs with near-infrared (NIR) absorption and emission as well as hydrophobic characteristics that could identify and kill Gram-positive bacteria efficiently (**Figure 4A**) (Zhao et al., 2022c). Antibacterial activity measurement showed that the CNDs could inactivate 99.99% of the Gram-positive bacteria (*S. aureus*, *E. faecalis*, and methicillin-resistant *S. aureus*) under 660 nm light irradiation for 5 min, while for the Gram-negative bacteria, the bactericidal efficiency was lower than 50%. Intracellular ROS detection and membrane potential measurement indicated that the antibacterial mechanism could probably be due to the ROS generated on the surface of bacteria membranes under excitation of 660 nm light, owning to the photoelectron conversion capabilities caused by their NIR absorption feature, as well as the hydrophobic interaction between the hydrophobic groups and Gram-positive bacteria membranes (**Figure 4C**). The scanning electron microscope (SEM) images of damaged cell membranes confirmed such hypotheses (**Figure 4B**).

CDs are also applied as adjuvant materials to improve the photocatalytic performance of PSs (Pan et al., 2014). Nano-semiconductor materials with unique photoinduced electron-hole pairs that can generate free radicals under the excitation of specific wavelengths of light have become a hot topic in the disinfection field (Li et al., 2017; Bagchi et al., 2018; Yılmaz et al., 2019). However, the low photocatalytic efficiency and the narrow emission spectrum restricted their clinical applications (Hou et al., 2015). Han et al. introduced *N*-doping carbon quantum dots (CQDs) into the lamellar MoS_2_ to improve the photocatalytic property of nano-semiconductor materials (Han et al., 2020). They coated the MoS_2_/CQDs hybrids on a Ti sheet through a spin-coating method as a potential antibacterial implant coating. Compared with Ti-PEI-MoS_2_, the Ti-PEI-MoS_2_/CQDs showed a slightly higher light absorbance but two times more ROS production. The enhanced photocatalytic property could be owning to the fact that the added CQDs acted as the assistant electron transporter, leading to the hindered recombination of the photogenerated electrons and holes (**Figure 4F**). According to the electrochemical impedance spectroscopy (EIS) results, Ti-PEI-MoS2/CQDs had the lowest impedance, followed by Ti-PEI-MoS2, Ti-PEI, and Ti-PEI. The mechanism was confirmed by the photocurrent experiments and EIS with the result of the highest electron conductivity obtained (including the most electrons production under light irradiation and the smallest impedance) among other groups. Therefore, the Ti-PEI-MoS_2_/CQDs mixture exhibited an efficient antibacterial effect towards representative bacterial model *S. aureus* and *E. coli* under 20 min of 660 nm light irradiation, indicating its possible application in implant coating to preventing peri-implantitis.

Another drawback of nano-semiconductor materials is that most of them could only utilize light in the ultraviolet or near-ultraviolet regions. The low visible light availability and the possible carcinogenicity of ultraviolet to oral tissues are two main barriers to their wide applications (Zhang et al., 2015; Kumar et al., 2018). An example of taking advantage of the up-conversion characteristics of CQDs to enhance the visible light utilization of PS was reported recently (Zhang et al., 2018). Aiming to resolve the enamel demineralization issue of the orthodontic brackets caused by surrounding bacteria (mostly *S. mutans*), Zhang et al. synthesized CQDs with an up-conversion fluorescence feature through the hydrothermal method. They prepared an antibacterial bracket with CQDs and ZnO dual layers via the sputtering method (**Figures 4D, E**). UV–vis absorption spectra showed an enhanced absorption of ZnO/CQDs coated brackets in the visible light region, indicating a greater band gap than that of ZnO. Moreover, the photodynamic antibacterial activity of the brackets was confirmed by colony-forming unit (CFU) assays, which showed that CQDs significantly improved the antibacterial effect of the bracket against common oral pathogens such as *S. mutans*, *S. aureus*, and *E. coli*.

### CDs in PTT

PTT is a process that converts light energy into heat by PTAs without oxygen involved. PTAs are crucial to PTT for their photothermal conversion capability, and CDs with a strong light absorption capacity and a broad absorption spectrum are excellent PTAs (Chen et al., 2020; Li et al., 2021a). For example, CDs synthesized from ascorbic acid could generate heat under NIR light irradiation at 808 nm (Sattarahmady et al., 2017). Therefore, the CDs could exsert the bactericidal properties against *S. aureus* by damaging the cell wall and increasing cell membrane permeability, resulting in protein leakage and endogenous ROS generation.

Moreover, CDs could exsert the combined antibacterial effect of PDT and PTT in the same antibacterial system. For instance, He et al. synthesized CDs incorporated from L-tryptophan and chlorhexidine acetate into TiO_2_ nanorods to improve the phototherapeutic efficiency of titanium implants (He et al., 2022). The CDs-doped TiO_2_ nanorod array (C-TiO_2_ NR) exhibited an excellent antibacterial property against *S. aureus* under the co-irradiation with 660 nm visible light and 808 nm NIR light. In this system, CDs not only assisted in increasing the photocatalytic efficiency of TiO_2_ for ROS generation but also absorbed NIR and exhibited hyperthermia capability.

### CDs for delivery of antimicrobial agents

CDs have a high surface area, high drug loading rate, good water solubility, and high biocompatibility, which make them carriers for the delivery of antimicrobial agents such as antibiotics and drugs for controlled release (Varghese and Balachandran, 2021). Studies have also demonstrated that modified CDs could enhance the antibacterial effects of antibiotics. For instance, CDs synthesized from gum arabic were conjugated with ciprofloxacin (Cipro@C-dots conjugate), displaying enhanced antimicrobial activity against Gram-positive and Gram-negative bacteria (Thakur et al., 2014). The conjugate could achieve a controlled release of ciprofloxacin as a solution to bacterial resistance caused by a high concentration of antibiotics.

In another work, Jijie et al. synthesized amine-terminated CDs (CDs-NH_2_) with citric acid and ethylenediamine as precursors (Jijie et al., 2018). The CDs-NH_2_ were then covalently functionalized with ampicillin (AMP) to produce CDs-AMP nanostructures to deliver AMP. The CDs-AMP conjugate showed effective antibacterial activity against *E. coli* attributed to its enhanced affinity binding onto the bacterial cell wall and thus higher exposure to ampicillin, as well as the intrinsic photodynamic capacity of CDs.

Antibiotic-carrying CDs can be used as nano-carriers to resist not only planktonic bacteria but also biofilms. Huang et al. innovatively incorporated CQDs into poly lactic-co-glycolic acid (PLGA) nanoparticles using microfluidic methods (Huang et al., 2020). The prepared CQD-PLGA composite nanoparticles had good loading capacity for azithromycin (AZI) and had stimuli-responsive release under laser irradiation. The outcomes demonstrated that the AZI-loaded CQD-PLGA hybrid nanoparticles had a chemical-photothermal synergistic effect on the *Pseudomonas aeruginosa* biofilm.

The overuse of traditional antibiotics and antimicrobial agents causing bacterial resistance is an increasing concern. Therefore, it is essential to prepare non-antibiotic antibacterial materials. Hakimeh et al. used GQDs as a linker to stabilize silver nanoparticles (AgNPs) synthesized *in situ* on cotton pads to prepare antibacterial pads (Teymourinia et al., 2021). Acted both as linkers and stabilizing agents, the GQDs can significantly enhance the antibacterial effects of AgNPs.

To further simplify the synthesis process and improve the antimicrobial properties, Wei et al. constructed a composite consisting of CDs and AgNPs (CDs/AgNPs) (Wei et al., 2021). Fluorescent CDs were produced by pyrolysis from natural *Gynostemma*. They could function as reducing and stabilizing agents in hazardous compounds. The complex was biocidal against both Gram-negative *E. coli* bacteria and Gram-positive *S. aureus* bacteria, indicating that CDs/AgNPs composites have great potential for future antibacterial and biomedical applications.

### CDs against oral intracellular bacteria

Bacteria that cause oral infections not only could exist in a planktonic form but can also invade the oral host cells and persist as intracellular bacteria. For instance, the primary pathogen of periodontitis, *P. gingivalis*, is well established to internalize into the oral epithelium cells, escaping the host immune response and acquiring antibiotic resistance (Xiong et al., 2012; Ye et al., 2017). Their persistence leads to the recurrence of infection (Miramoth et al., 2012). However, the commonly used antibiotics have limited penetrations into cells, which requires us to develop novel intracellular antibacterial drugs (Abed and Couvreur, 2014).

Studies have shown that CDs have good biocompatibility and can be internalized into cells. For example, Li et al. synthesized aggregated CDs (ACDs) specifically located in the lysosomes of macrophages (Li et al., 2022b). The ACDs assisted in digesting bacteria delivered into the lysosome, stressing an enhanced bactericidal ability. Other studies revealed the anti-inflammatory ability, and enhanced immunomodulatory activity of CDs internalized into macrophages (Li et al., 2018b; Yavuz et al., 2020). Donãate-Buendia et al. confirmed that CDs could be internalized into oral epithelial cells within 10 min of incubation (Doñate-Buendia et al., 2018). The studies above provide a prerequisite for the application of CDs against oral intracellular bacteria.

**Figure 5:**
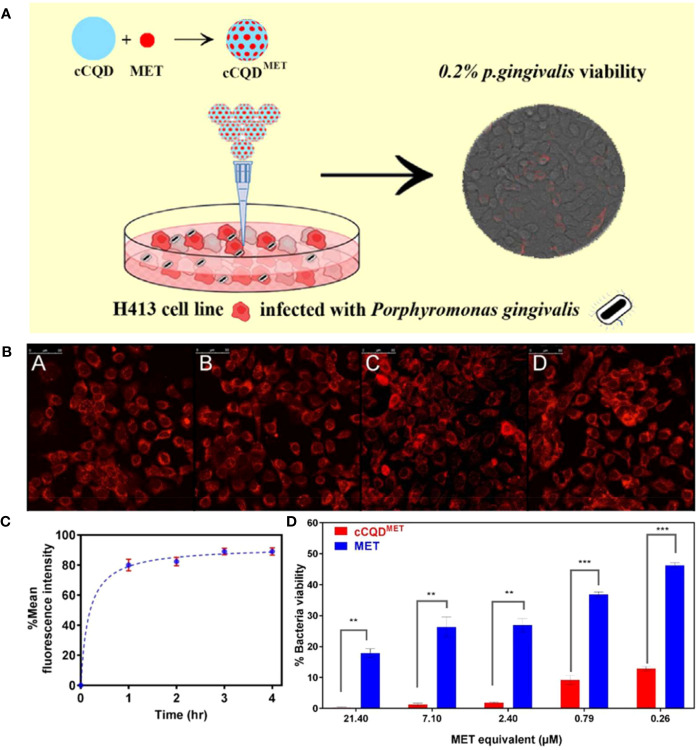
**(A)** Schematic diagram of cCQD^MET^ killing intracellular *P. gingivalis*. **(B)** Cellular uptake of cCQD^MET^ with increasing fluorescence intensity of internalized cCQD^MET^ over a 4-h period: (A) 1h, (B) 2h, (C) 3h and (D) 4h. **(C)** The mean fluorescence intensity of the internalized cCQD compared to control wells show plateauing over a 4-h period. **(D)** Summary of average counting numbers of intracellular *P. gingivalis* colonies (CFU) to indicate the efficacy of cCQDMET conjugate; Legend: paired t-test (mean ± SD, two-tailed, 95% confidence interval) from at least 3 consecutive experiments. **=(p<0.01) and ***=(p<0.001) (Ardekani et al., 2019). Copyright Elsevier reprinted with permission. License Number: 5534220750148.

Therefore, a recent study used CDs as a nanocarrier to transport antibiotics into oral epithelial cells for combating intracellular *P. gingivalis* (Ardekani et al., 2019). Ardekani et al. constructed a nano-antibiotic conjugate with CQDs derived from chlorophyll (cCQD) to serve as a drug carrier and the standard antibiotic metronidazole (MET) loaded on its surface for the intracellular bactericidal purpose (cCQD^MET^). The drug payload of cCQD was 80% (w/w), and the particle size was controlled to range from 2 to 4 nm for consistent internalization and intracellular MET delivery, resulting in an uptake rate of 90% within 3 h of the incubation. An intracellular *P. gingivalis* infection model was constructed to evaluate the intracellular microbicidal activity of the conjugate. Compared with MET alone, a 72% enhancement of the intracellular microbicidal activity of the conjugate at concentrations as low as 0.26 mM MET equivalent was observed (**Figure 5**). This work suggests a promising ability of CDs against oral intracellular bacteria, which also provides new insight into preventing the recurrence of oral infection. However, research on this application is still at an early stage, and further optimization of the materials is expected.

### CDs against oral biofilms

Most oral flora grows in bacterial biofilm, the leading cause of oral infectious diseases such as dental caries, pulpitis, periradicular lesions, and periodontitis (Larsen and Fiehn, 2017). The dynamic process of biofilm formation is generally categorized into three steps: the formation of the acquired pellicle, bacterial adhesion and aggregation, and maturation of the biofilm (Rickard et al., 2003; Sheng et al., 2022).

Pathogenic biofilms are widely considered a distinguishing factor leading to antibiotic resistance, attributed to their composition and intricate structure (Hu et al., 2019). The reason could attribute to the formation of EPS barriers, the complex multi-community gradient distribution, and the diverse intercellular communication among the ordered bacterial communities (Hu et al., 2019; Makabenta et al., 2021).

Apart from the mechanisms mentioned above, oral biofilms have unique properties that can further reduce antimicrobial efficacy. First, considering that most oral biofilms are infiltrated in saliva and gingival crevicular fluid, the fluid environment and oral hygiene practices dilute the concentration of antibiotics (Hu et al., 2019). Second, the presence of saliva and food increases the concentration of EPS and thus makes the penetration of antimicrobial agents more difficult. In addition, the acidic environment of cariogenic biofilms could further promote the synthesis of EPS, reducing the antibacterial effect to a great extent (Bowen and Koo, 2011; Afrasiabi et al., 2021). Therefore, novel materials with better penetration ability and the capacity to inhibit or destroy EPS are in great demand.

As a novel nanomaterial with good antimicrobial properties, CDs could inhibit biofilm formation from multiple aspects. For example, GQDs with graphite as precursors were coupled with curcumin (Cur) to prepare photoexcited graphene-derivative-based drug nanocomposites (GQD-Cur) (Pourhajibagher et al., 2019). Perio-pathogen mixed biofilms containing *A. actinomycetemcomitans*, *P. gingivalis*, and *P. intermedia* were constructed for antibiofilm investigations. Results showed that GQD-Cur increased ROS generation under blue light-emitting diode exposure, indicating a high inhibitory effect on mixed biofilms via aPDT. Moreover, the qRT-PCR analysis showed that the pathogenic gene expression profile relating to biofilm formation (rcpA, fimA, and inpA) was remarkably downregulated, therefore suppressing the progression of biofilm formation.

**Figure 6:**
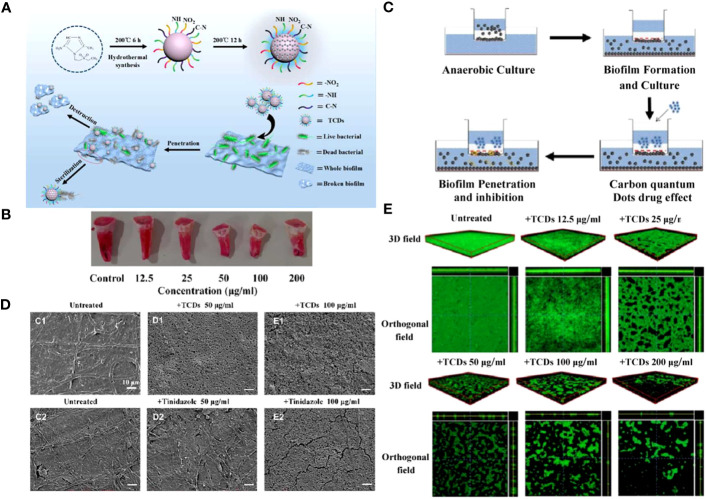
**(A)** Schematic diagram of specific anti-biofilm activity of TCDs. **(B)** *In vitro P. gingivalis* adhesion tooth models. **(C)** Schematic diagram of biofilm penetration sterilization experiment. **(D)** SEM images of *P. gingivalis* biofilm treated with different concentrations of TCDs or tinidazole. **(E)** 3D and orthogonal fields of the inhibitory effects of TCDs on *P. gingivalis* biofilm formation (Liang et al., 2020a). Open access article with no copyright permission.

In addition to gene regulation or destruction, hydrophilic CDs also play a role in cellular adhesion and aggregation phage. As is known to all, the hydrophobicity of bacteria contributes to its adhesion to host cells and other abiotic surfaces (Cozens and Read, 2012). Liang et al. synthesized hydrophilic tinidazole CQDs (TCDs) through a hydrothermal method(Liang et al., 2020a). They found that TCDs significantly increased the hydrophilic properties of *P. gingivalis* compared to its precursor tinidazole and reduced bacterial adhesion on the surface of tooth models. Moreover, the excellent permeability of TCDs enabled them to go deep into the lower layers of biofilms, killing the hidden bacteria and exhibiting a robust destructive ability of mature biofilms (**Figure 6**).

The higher concentration of EPS in oral biofilms results in smaller pores and stronger resistance to antimicrobial agents. Research showed that particles with diameters ranging between 2 and 10 nm are more beneficial for their diffusion in biofilms (Peulen and Wilkinson, 2011). Cariogenic biofilm-targeted nanocomposites were designed to disrupt mature biofilms of *S. mutans* (Ostadhossein et al., 2021), consisting of positively charged CHX PR_4_+ CDs and a pH-responsive layer of an amphiphilic polymer. The nanoparticles expressly released CDs in the acidic environment of the *S. mutans* biofilms, enabling CDs to penetrate the deep layer of the biofilms through their hyperpermeability and kill the deep *S. mutans*. More importantly, such targeted materials did not interfere with the rest of the oral microbes, maintaining the balance of the oral flora, which has important implications for preserving the function of oral flora against foreign pathogens.

### CDs against fungi

Fungi is one of the main pathogenic microorganisms in the oral cavity (Naglik et al., 2013). Its excessive aggregation can lead to oral candidiasis, a highly recurrent and prevalent infection, especially in immunocompromised individuals (Patil et al., 2015). A primary pathogen of the fungal infection is *C. albicans* (Swidergall and Filler, 2017). This opportunistic fungal pathogen could invade oral epithelial cells by induced endocytosis and active penetration, resulting in increased difficulty in the clinical treatment of oral candidiasis (Montes and Wilborn, 1968).

**Figure 7:**
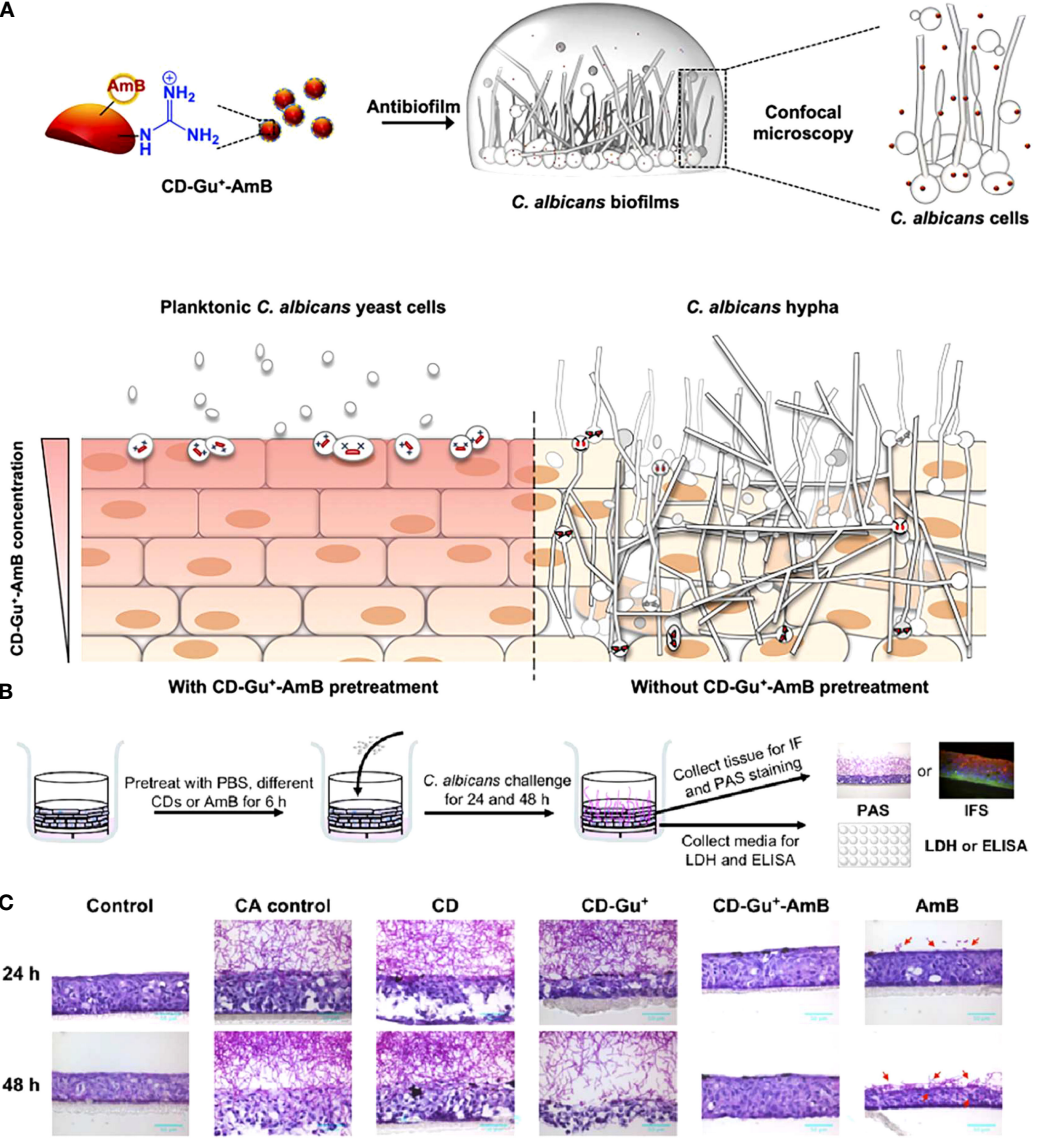
**(A)** Schematic diagram of the antibiofilm effect of CD-Gu^+^-AmB and their protection of reconstituted human oral epithelia (RHOE) from the invasion of *C. albicans*. **(B)** Schematic diagram of the experimental design of CD-Gu^+^-AmB-Embedded RHOE against invasion by *C. albicans*. **(C)** The cryosection of the collected tissue samples was performed with periodic acid-Schiff (PAS) (Li et al., 2019). Copyright ACS reprinted with permission.

However, owing to their poor bioavailability and dose-dependent toxicity, the application of frontline antifungals is restricted (Liu et al., 2017b). Li et al. developed a CD-based theranostic nanoplatform (CD-Gu^+^-AmB) for perturbing the intracellular location of *C. albicans*, as well as tracking the penetration of antifungals (**Figure 7A**) (Li et al., 2019). Water-soluble red-emissive carbon dot (CD) cores were synthesized with citric acid and ethylenediamine as the precursor. They were decorated with positively charged guanidine groups (Gu^+^) and functionalized with a clinical "gold standard" antifungal polyene (amphotericin B, AmB). The generated nanoplatform preserved the antifungal effect of AmB, hence performing excellent antifungal effects in both planktonic and biofilm forms. A three-dimensional organotypic human oral epithelial tissue model was established to assess the interactions of CD with oral epithelial compartments, and the results showed that CD-Gu^+^-AmB could resist the invasion of *C. albicans* to a great extent through extra- and intracellular accumulation, forming a "shielding" layer throughout the tissue (**Figures 7B, C**).

Most manifestations of candidiasis are related to Candida biofilm formation (Ramage et al., 2001). It is well known that the biofilm formation process progresses in three phases. The early stage manifested as surface adhesion and cellular aggregation of *C. albicans* cells in yeast forms. During the intermediate phases, the hyphae form of *C. albicans* appeared, and microcolonies were covered with noncellular material composed mainly of polysaccharides. The amount of noncellular material increased to form a three-dimensional architecture, suggesting the arrival of a maturation phase (Chandra et al., 2001; Jabra-Rizk et al., 2004; Kojic and Darouiche, 2004).

Regarding Candida infections on the surface of prostheses and implanted devices such as denture stomatitis and peri-implantitis, surface adhesion, and filamentation are commonly believed to be critical in biofilm formation (Chandra et al., 2001; Mima et al., 2012; Shaikh et al., 2018; Alrabiah et al., 2019). Such infections are often refractory to clinical antifungal agents attributed to similar drug-resistant mechanisms with bacterial biofilms (Kojic and Darouiche, 2004). Confronted with the above issues, Shaikh et al. used fruit juice extracted from Citrus limetta as the precursor to prepare colloidal luminescent CDs (C-dots) by solvothermal method (Shaikh et al., 2018). They incubated them for 24 h with *C. albicans* adhering to a polystyrene surface. Results showed that the generated C-dots could eliminate 40% of the biofilms at a concentration of 75 μg mL^-1^. An absence of true hyphae was observed, indicating that CDs interfered with biofilm formation by inhibiting the adhesion of *C. albicans*, as well as the inhibition of filamentation.

Overall, CDs could exhibit excellent antimicrobial capacity through multiple mechanisms and possess the ability to act as novel oral antimicrobial agents. They have broad prospects in the application of anti-oral infectious diseases.

## Microorganism-imaging abilities of CDs

**Figure 8:**
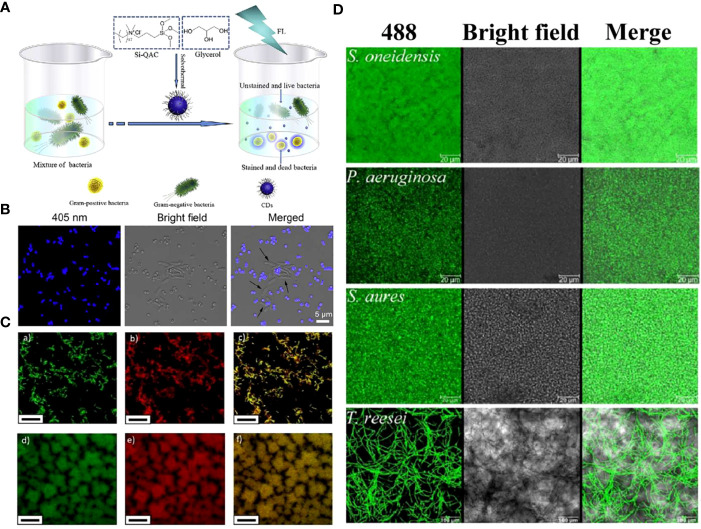
**(A)** Schematic illustrating the preparation of quaternized CDs and their application for selective imaging and killing of Gram-positive bacteria. **(B)** Confocal images of the mixture of *E*. *coli* and *S. aureus* cells after incubation with quaternized CDs (Yang et al., 2019). Copyright Elsevier reprinted with permission. License Number: 5534631145702. **(C)** Multi-color fluorescence confocal microscopy images of (a-c) *S. mutans* and (d-f) *P. gingivalis* excited at (a, d) 488 nm and (b, e) 534 nm, (c, f) and the merged images, respectively (Zhao et al., 2019). Copyright RCS reprinted with permission. Order License ID: 1347827-1. **(D)** Biofilms of different microorganism species, including the model biofilm bacteria *S. oneidensis*, *P. aeruginosa*, *S. aureus*, and fungus *T. reesei*, were stained by CDs-605 (Lin et al., 2017). Copyright RCS reprinted with permission. Order License ID: 1347830-1.

The fluorescence characteristics and low toxicity of CDs make them new microbial fluorescence imaging agents, showing the high potential for application in microbial detection, bacterial identification, and biofilm imaging areas (Ritenberg et al., 2016; Das et al., 2017; Hua et al., 2017; Das et al., 2018; Lin et al., 2019). For example, Yang et al. constructed quaternized CDs with bacterial contact-enhanced fluorescence emission properties (Yang et al., 2019). The quaternized CDs enabled selective inactivation and identification of Gram-positive bacteria, which could be more convenient and faster for Gram-type differentiation, thus leading to a better diagnosis of infection (**Figures 8A, B**). Liu et al. studied and explored that carbon dots doped with N and Cl elements showed good recognition and selectivity for gram-positive bacteria (Liu et al., 2021b). Positively charged N, Cl-CDs could not only distinguish gram-positive bacteria through selective fluorescence imaging but also have an antibacterial effect on them.

In another study, Zhao et al. synthesized acidophilic dual-emission fluorescent carbonized PDs (R-CPDs, R/B-CPDs, B-CPDs) capable of selectively detecting common oral pathogens such as *S. mutans* and *P. gingivalis* (Zhao et al., 2019). The unique acidophilic CDs also allowed for stable imaging under acidic conditions of oral cariogenic biofilms, providing new possibilities for alternative traditional methods in oral microbial detection (**Figure 8C**).

Plaque-derived infections are quite common among all oral infections (Kolenbrander et al., 2010). Along with the progress of anti-oral biofilm scientific research and biofilm diagnostics, CDs with characteristics such as biosecurity, stability, and ultra-small size became an ideal biofilm stain. It has been demonstrated that CDs could image both Gram-positive and negative bacterial biofilms (Ritenberg et al., 2016; Ran et al., 2019). Further, CDs derived from *L. plantarum* LLC-605 (CDs-605) could easily pass through the EPS barrier and penetrate deeply, imaging biofilm-encased microorganisms, including Gram-positive bacteria (*S. aureus*), Gram-negative bacteria (*E. coli*, *Shewanella oneidensis*, and *Pseudomonas aeruginosa*), and fungus (*Trichoderma reesei*) (**Figure 8D**) (Lin et al., 2017). Zhao et al. prepared QCS-EDA-CDs, which have been successfully used for *S. aureus* and its biofilm imaging due to their excellent optical properties (Zhao et al., 2022b). This application of CDs provides a powerful aid for the mechanistic understanding of biofilm formation and diffusion processes.

CDs have a wide range of microbial detection and imaging capabilities, playing an early diagnosis role in infectious diseases, and have a good application prospect in the auxiliary diagnosis of clinical periodontitis, apical periodontitis, and other oral infections. It provides a simpler, faster, and more accurate method of microbiological examination for patients with severe disease or poor response to conventional treatment. By detecting the dominant pathogens, CDs could help to screen drugs, evaluate their efficacy, and monitor the maintenance period of treatment. They also provide new ideas for real-time tracking of the mechanism of oral pathogenic bacteria on diseases. In the current literature, researchers have also proposed prospects for applying microbial imaging capabilities of CDs in the oral cavity. Ostadhossein et al. studied the reduction of oral biofilm formation by engineering cluster CDs nanoparticles without destroying the oral balance of nature (Ostadhossein et al., 2021). In this article, they envisioned a self-assembled structure with high surface functional group density. This structure can be introduced into the targeting part by simple chemical methods in the future. It also has great prospects to be applied to the sensitive detection and molecular imaging of biofilms using CDs luminescence technology. As for oral fungal infection, Li et al. developed a therapeutic nano platform (CD-Gu^+^-AmB) to track the penetration of antifungal drugs and interfere with the invasion of *C. albicans* to oral epithelium through bioimaging (Li et al., 2019). CDs were proposed as a promising fluorescent agent and an ideal probe for *in vitro* and *in vivo* biological imaging. It can be observed through imaging that CDs with rich amines or positively charged groups exhibit typical accumulation in nucleoli, thus evaluating the intracellular internalization and potential cytotoxicity of CDs.

## Clinical translation and limitations

In the past decades, carbon nanomaterials have shown great potential for clinical translation for excellent properties such as proper size, good biodegradability, and biosafety (Soltani et al., 2020; Fan et al., 2021). Noteworthy, carbon nanoparticles suspension injection (CNSI) has been approved by the China Food and Drug Administration (CFDA) for the clinical application of lymphatic tracers, assisting over 100 000 patients each year (Wang et al., 2020).

As an emerging carbon nanomaterial, CDs have also been put into the study in anti-tumor, antibacterial, tissue repair and other fields (Feng et al., 2016; Li et al., 2020a). However, in the field of clinical translation for disease diagnosis and treatment, research on CDs is still in its infancy.

Take cancer therapy as an example, most research stays in the 2D cell culture and animal models stage (Gong et al., 2019; Boobalan et al., 2020; Pang et al., 2020). In recent years, studies that combine CDs with organ-on-a-chip platforms have gradually increased to better emulate the functionality and composition of human organs, most of them showing inspiring results.

A typical example was a 3D breast-cancer-on-chip platform constructed for the evaluation of a CDs-based drug delivery system (Chen et al., 2018). Results indicated that the CDs loaded with doxorubicin could be transported across an endothelial monolayer rapidly and were nontoxic to the cells, capable of monitoring dynamic transport behavior and assessing cytotoxicity in the same system. In another study, red-emissive biotinylated CDs were loaded with irinotecan for targeted guidance, bioimaging, and effective anticancer activity (Scialabba et al., 2019). The properties were confirmed by 3D patient organoids (tumor-on-a-dish preclinical models) of primary breast cancer cells from human biopsies to predict drug response and metastatic potential of human tumors.

Similar research on CDs acting on human pathological samples has also been reported for oral infectious diseases. In a study by Tang et al., fucoidan-derived CDs were prepared as root canal disinfectants during root canal therapy (RCT) for the complete removal of *E. faecalis* biofilms in patients with persistent endodontic infections (Tang et al., 2022). After co-incubation with *E. faecalis*-infected dentin blocks, CDs greatly eliminated bacteria on the root canals and in the dentinal tubules, exhibiting excellent potential for clinical applications. While in another study, Li et al. employed engineered commercialized oral epithelium tissues to evaluate penetration and exfoliation profiles of CD-based antifungal platforms and the ability of platforms against fungal invasion (Li et al., 2019).

CDs in different forms have been prepared to increase the application diversity in the oral cavity for purpose of treating various oral infectious diseases. The most common ones are antibacterial nanomedicine, root canal disinfectants, antibacterial coatings on the surface of implants, and coatings on orthodontic brackets (Liu et al., 2017a; Han et al., 2020; Taufiq et al., 2020; Tang et al., 2022). It has been reported that CDs can cross-link with polysaccharides to form antibacterial hydrogels by virtue of a large number of surface chemical groups, thereby obtaining flexible injectability and self-healing properties (Yang et al., 2021).

Bactericidal efficiency is a key consideration for nanomedicines. To pursue a more efficient sterilization effect, researchers began to combine CDs with other antibacterial agents to design a multiple or synergistic antibacterial platform (Dong et al., 2018; Zhang et al., 2018; Pourhajibagher et al., 2019). For example, Chu et al. constructed a near-infrared CD-based platform with bioimaging as well as synergistic phototherapy and quaternary ammonium bactericidal abilities (Chu et al., 2021). They first combined NIR-emitting CDs (RCDs) and transition metal ions (Cu) to form Cu-doped RCDs (Cu-RCDs) for enhancement of light absorption. The quaternized Cu-RCDs (Cu-RCDs-C_35_) were synthesized by conjugating Cu-RCDs with a quaternary ammonium compound, cocoamidopropyl betaine (CAB-35). This synergistic therapeutic strategy, including quaternary ammonium compounds, PDT and PTT, increased the bactericidal efficiency of the nanoplatform against *S. aureus* and *E. coli* to over 99%. The work suggests that synergistic therapeutic strategies should be more considered in designing oral antibacterial materials to improve the therapeutic effect.

However, before the antibacterial CDs can be used in the clinical diagnosis and treatment of oral microbial infections, the following aspects need to be considered. First, it is still necessary to understand clearly the antibacterial mechanism of CDs against oral microorganisms for the better design of multifunctional antibacterial platforms and to improve their performance by adjusting the residence time, permeability, and sterilization rate of drugs against the oral pathogenic microbe.

Second, for the multiple microorganisms in the oral cavity, CDs should be able to remove pathogenic bacteria while keeping the normal oral flora unaffected. More work can be put into pursuing CDs capability of regulating the structure of oral flora and metabolic environment, and treating recurring oral infections caused by dysbacteriosis. To achieve this, new targeted nanomaterials should be designed. For instance, researchers took advantage of the acidic characteristics of oral pathogenic biofilms to design pH-responsive nanocomposites, which can release CDs around the cariogenic acidic biofilms in a targeted manner, ensuring that the normal oral flora is not affected (Ostadhossein et al., 2021). While CDs targeting dominant pathogenic bacteria and critical virulence factors in oral pathogenic biofilms [virulence genes (Hu et al., 2019)] have not been reported. The regulation of CDs on oral flora and metabolic environment has also not been reported so far. However, it was confirmed that semi-carbonized nanodots (SCNDs) extracted from charred herbs can regulate the structure of intestinal flora by reducing stress-related excessive neuroendocrine response (Lu et al., 2021). The study provides a new idea for research on regulating oral flora by CDs.

Third, it is essential to determine the interaction between CDs and saliva. Liu et al. reported copper-doped carbon dots (Cu-CDs) that display enhanced catalytic activity in the oral environment for inhibiting *S.mutans* adhesion and for biofilm eradication (Liu et al., 2022b). The stability of Cu-CDs in the oral environment was tested by exposing Cu-CDs to saliva at different pH values, and the result indicated that the threat of copper ion release was negligible. They also cultivated oral biofilm on the saliva-coated human teeth, and an improved flow system designed to simulate saliva flow was used to add bacteria for biofilm incubation and add Cu-CDs for simulation of the bactericidal process in the oral environment.

However, in the oral fluid environment, the saliva enriched with proteins could affect the antibacterial and bio-imaging abilities of the suspended CDs by affecting aspects such as adsorption and aggregation. Few works have been done to explore this question. There are researches on the interaction of saliva and other antibacterial nanomaterials that support this point of view (Xu et al., 2010; Besinis et al., 2015; Pinďáková et al., 2017). For instance, Pokrowiecki et al. verified that saliva could affect the stability and behavior of nano-formulation mixtures based on different compositions of Ag nanoparticles and ZnO NPs (Pokrowiecki et al., 2019). A rapid agglomeration process was indicated as the nanoparticles were introduced to the saliva. It is estimated that this phenomenon was possibly related to electronic interaction, the formation of the protein corona coated on the surface of particles, and the formation of hydrogen bonds. Therefore, we conjecture that CDs with different surface modifications may also interact with saliva or proteins in it, and it is recommended for future studies to further explore the specific mechanisms and principles of the interaction process.

Fourth, the safety of the CDs remains to be considered. Nanomaterials with diverse surface status usually have different effect on cells, and current studies on the biocompatibility of CDs have reached different conclusions (Zhou et al., 2018a; Zhou et al., 2018b). In most experiments, CDs are proven to be biocompatible. Synthesized copper-doped carbon dots (Cu-CDs) were co-cultured with skin fibroblasts L929 cells and human dental pulp cells (hDPC) (Liu et al., 2022). The result indicated that L929 cells and hDPC showed good biocompatibility on both CDs and Cu-CDs. At the same time, researchers demonstrated that Cu-CDs have short blood circulation and can be excreted through urine and feces, thereby reducing accumulation in major organs, further validating the safety of Cu-CDs. However, other researchers found out that CDs may have potential safety risks. For example, Jia et al. demonstrated that positively charged PL-CDs have the possibility of resulting in intestinal flora dysbiosis via inhibiting probiotic growth and simultaneously activating intestinal inflammation (Jia et al., 2023). They are verified to limit the activity of intestinal epithelial cells *in vitro* and could result in damage to the intestines. In another example, Liu et al. demonstrate that CDs derived from glucose breakdown into compounds hazardous to healthy human cells under low light exposure, indicating that light exposure or other factors should be considered in future assessments of the safety of CDs (Liu et al., 2021c).

## Conclusion and outlook

There has been a growing tendency to use CDs for the treatment of oral microbial infections in recent years. In this review, we introduced the application of CDs against oral microorganisms from the perspective of antimicrobial mechanisms to provide more possibilities to help overcome theoretical hurdles for the clinical adoption of CDs for treating oral pathogenic infectious. In addition to their ability to efficiently killing oral plankton pathogenic microorganisms, CDs could be internalized into oral epithelial cells to kill intracellular bacteria. Meanwhile, CDs could exhibit anti-biofilm ability by inhibiting their formation and destroying mature biofilms, offering a considerable advantage in addressing the oral microbial drug-resistance issue. Furthermore, the optical characteristics of CDs also enable fluorescence imaging of oral microorganisms, which is of great significance for the early diagnosis of infections and scientific research. Finally, we summarize their potential and limitations in clinical translation.

Nevertheless, still more effort is needed to put into the optimization of CDs from the following aspects. First, most studies mentioned above are limited to common pathogenic bacteria in the oral cavity, e.g., *P. gingivalis*, *S. mutans*, *S. aureus*, and their single-species biofilms. However, clinical oral infectious diseases are caused mainly by the combined effect of various pathogenic microbes and the formation of multi-species plaque biofilms. As such, studies on the effect of CDs on other oral pathogens and oral pathogens mixed biofilms are needed to simulate clinical oral microbial infectious.

Second, CDs that process the capability of targeting pathogenic bacteria or biofilms need to be developed, constructing a precise CDs-based platform for antibacterial treatment. It is necessary to target and selectively kill specific microbial strains in the complex biofilm network and maintain the microecological balance of oral flora while achieving thorough treatment.

Third, the impact of CDs on the structure of oral flora and the metabolic environment of biofilm should be further explored. We expect that CDs could modulate the microecology of the oral flora, return the flora under pathological conditions to a dynamic balance to treat oral microbial infectious diseases, and prevent secondary infection after treatment.

Fourth, research on CDs can be combined with intelligent technologies to optimize the synthetic method of CDs, and to obtain CDs with more functionalities in response to the treatment of complicated oral diseases. Traditional methods of synthesizing CDs mostly rely on experience and lack data statistics. Therefore, future studies on CDs can take advantage of computer simulation and machine learning technology to establish different models by analyzing data such as precursors, synthesis routes, physicochemical properties, and therapeutic effects of existing CDs. By this a parametric and standardized data platform can be constructed, with which researchers are able to design and obtain ideal antibacterial CDs by screening precursors and functionalized modifiers, optimizing synthetic routes, and predicting properties of potential structures, avoiding unexpected side effects and unnecessary waste of materials. In addition, to complete the precise behavior of antibacterial CDs in the microstructure of the human oral cavity, an integrated platform can be established for antibacterial and imaging. Besides, it is essential to establish a platform for antibacterial imaging and develop a complete real-time imaging tracking and monitoring system to realize drug delivery and antibacterial effect evaluation guided by *in vitro* imaging. CDs with excellent optical properties could also be used for early diagnosis of infection through non-invasive fluorescence imaging technology.

Fifth, in terms of clinical translation and popularization, toxicology studies of CDs on large animals and even primates are necessary to be conducted. In addition, functional research on 3D organotypic models of oral tissues is helpful for the evaluation of the antibacterial effect of CDs in the actual oral environment. With the advancements of the challenges above, we believe that CDs will find increasing applications in the field of therapy and diagnosis for oral microbial infections.

## Author contributions

YJ, JM and XW: Wrote the manuscript. CY and XW: Searched literatures. YJ, CY and JM: Illustrated figures and tables. TW, GL and QZ: Edited the manuscript. QZ: Designed and supervised the review. All authors contributed to manuscript revision, read, and approved the submitted version.

## References

[B1] Abed N. Couvreur P. (2014). Nanocarriers for antibiotics: A promising solution to treat intracellular bacterial infections. Int. J. Antimicrob. Agents 43, 485–496. doi: 10.1016/j.ijantimicag.2014.02.009 24721232

[B2] Afrasiabi S. Chiniforush N. Barikani H. R. Partoazar A. Goudarzi R. (2021). Nanostructures as targeted therapeutics for combating oral bacterial diseases. Biomedicines 9, 1435. doi: 10.3390/biomedicines9101435 34680553 PMC8533418

[B3] Aithal G. C. Nayak U. Y. Mehta C. Narayan R. Gopalkrishna P. Pandiyan S.. (2018). Localized in *situ* nanoemulgel drug delivery system of quercetin for periodontitis: Development and computational simulations. Molecules 23, 1363. doi: 10.3390/molecules23061363 29882751 PMC6099597

[B4] Allaker R. P. Memarzadeh K. (2014). Nanoparticles and the control of oral infections. Int. J. Antimicrob. Agents 43, 95–104. doi: 10.1016/j.ijantimicag.2013.11.002 24388116

[B5] Almonacid Suarez A. M. Zhou Q. van Rijn P. Harmsen M. C. (2019). Directional topography gradients drive optimum alignment and differentiation of human myoblasts. J. Tissue Eng. Regen. Med. 13, 2234–2245. doi: 10.1002/term.2976 31677226 PMC6973069

[B6] Alrabiah M. Alshagroud R. S. Alsahhaf A. Almojaly S. A. Abduljabbar T. Javed F. (2019). Presence of Candida species in the subgingival oral biofilm of patients with peri-implantitis. Clin. Implant Dent. Relat. Res. 21, 781–785. doi: 10.1111/cid.12760 30908836

[B7] Ardekani S. M. Dehghani A. Ye P. Nguyen K. A. Gomes V. G. (2019). Conjugated carbon quantum dots: Potent nano-antibiotic for intracellular pathogens. J. Colloid Interface Sci. 552, 378–387. doi: 10.1016/j.jcis.2019.05.067 31136856

[B8] Armitage G. C. Cullinan M. P. Seymour G. J. (2010). Comparative biology of chronic and aggressive periodontitis: Introduction. Periodontol. 2000 53, 7–11. doi: 10.1111/j.1600-0757.2010.00359.x 20403101

[B9] Bagchi D. Rathnam V. S. S. Lemmens P. Banerjee I. Pal S. K. (2018). NIR-light-active ZnO-based nanohybrids for bacterial biofilm treatment. ACS Omega 3, 10877–10885. doi: 10.1021/acsomega.8b00716 30320255 PMC6173506

[B10] Baker S. N. Baker G. A. (2010). Luminescent carbon nanodots: Emergent nanolights. Angew. Chemie - Int. Ed. 49, 6726–6744. doi: 10.1002/anie.200906623 20687055

[B11] Besinis A. De Peralta T. Tredwin C. J. Handy R. D. (2015). Review of nanomaterials in dentistry: Interactions with the oral microenvironment, clinical applications, hazards, and benefits. ACS Nano 9, 2255–2289. doi: 10.1021/nn505015e 25625290

[B12] Bing W. Sun H. Yan Z. Ren J. Qu X. (2016). Programmed bacteria death induced by carbon dots with different surface charge. Small 12, 4713–4718. doi: 10.1002/smll.201600294 27027246

[B13] Boobalan T. Sethupathi M. Sengottuvelan N. Kumar P. Balaji P. Gulyás B.. (2020). Mushroom-derived carbon dots for toxic metal ion detection and as antibacterial and anticancer agents. ACS Appl. Nano Mater. 3, 5910–5919. doi: 10.1021/acsanm.0c01058

[B14] Bowen W. H. Koo H. (2011). Biology of streptococcus mutans-derived glucosyltransferases: Role in extracellular matrix formation of cariogenic biofilms. Caries Res. 45, 69–86. doi: 10.1159/000324598 PMC3068567 21346355

[B15] Budimir M. Marković Z. Vajdak J. Jovanović S. Kubat P. Humpoliček P.. (2021). Enhanced visible light-triggered antibacterial activity of carbon quantum dots/polyurethane nanocomposites by gamma rays induced pre-treatment. Radiat. Phys. Chem. 185, 109499. doi: 10.1016/j.radphyschem.2021.109499

[B16] Chai S. Zhou L. Pei S. Zhu Z. Chen B. (2021). P-doped carbon quantum dots with antibacterial activity. Micromachines 12, 1116. doi: 10.3390/mi12091116 34577758 PMC8466419

[B17] Chandra J. Kuhn D. M. Mukherjee P. K. Hoyer L. L. McCormick T. Ghannoum M. A. (2001). Biofilm formation by the fungal pathogen Candida albicans: Development, architecture, and drug resistance. J. Bacteriol. 183, 5385–5394. doi: 10.1128/JB.183.18.5385-5394.2001 11514524 PMC95423

[B18] Chen Y. Gao Y. Chen Y. Liu L. Mo A. Peng Q. (2020). Nanomaterials-based photothermal therapy and its potentials in antibacterial treatment. J. Control. Release 328, 251–262. doi: 10.1016/j.jconrel.2020.08.055 32889053

[B19] Chen Y. Gao D. Wang Y. Lin S. Jiang Y. (2018). A novel 3D breast-cancer-on-chip platform for therapeutic evaluation of drug delivery systems. Anal. Chim. Acta 1036, 97–106. doi: 10.1016/j.aca.2018.06.038 30253842

[B20] Chu X. Zhang P. Wang Y. Sun B. Liu Y. Zhang Q.. (2021). Near-infrared carbon dot-based platform for bioimaging and photothermal/photodynamic/quaternary ammonium triple synergistic sterilization triggered by single NIR light source. Carbon N. Y. 176, 126–138. doi: 10.1016/j.carbon.2021.01.119

[B21] Cozens D. Read R. C. (2012). Anti-adhesion methods as novel therapeutics for bacterial infections. Expert Rev. Anti Infect. Ther. 10, 1457–1468. doi: 10.1586/eri.12.145 23253323

[B22] Cui F. Ye Y. Ping J. Sun X. (2020). Carbon dots: Current advances in pathogenic bacteria monitoring and prospect applications. Biosens. Bioelectron. 156, 112085. doi: 10.1016/j.bios.2020.112085 32275580

[B23] Das P. Bose M. Das A. K. Banerjee S. Das N. C. (2018). One-step synthesis of fluorescent carbon dots for bio-labeling assay. Macromol. Symp. 382, 1–6. doi: 10.1002/masy.201800077

[B24] Das P. Bose M. Ganguly S. Mondal S. Das A. K. Banerjee S.. (2017). Green approach to photoluminescent carbon dots for imaging of gram-negative bacteria Escherichia coli. Nanotechnology 28, 195501. doi: 10.1088/1361-6528/aa6714 28417900

[B25] Dewhirst F. E. Chen T. Izard J. Paster B. J. Tanner A. C. R. Yu W. H.. (2010). The human oral microbiome. J. Bacteriol. 192, 5002–5017. doi: 10.1128/JB.00542-10 20656903 PMC2944498

[B26] Ding X. Tang Q. Xu Z. Xu Y. Zhang H. Zheng D.. (2022). Challenges and innovations in treating chronic and acute wound infections: from basic science to clinical practice. Burn. Trauma 10, tkac014. doi: 10.1093/burnst/tkac014 PMC9123597 35611318

[B27] Doñate-Buendia C. Torres-Mendieta R. Pyatenko A. Falomir E. Fernández-Alonso M. Mínguez-Vega G. (2018). Fabrication by laser irradiation in a continuous flow jet of carbon quantum dots for fluorescence imaging. ACS Omega 3, 2735–2742. doi: 10.1021/acsomega.7b02082 30023850 PMC6044845

[B28] Dong X. Bond A. E. Pan N. Coleman M. Tang Y. Sun Y. P.. (2018). Synergistic photoactivated antimicrobial effects of carbon dots combined with dye photosensitizers. Int. J. Nanomed 13, 8025–8035. doi: 10.2147/IJN.S183086 PMC6267493 30568443

[B29] Fan Y. Liu Y. Zhou Q. Du H. Zhao X. Ye F.. (2021). Catalytic hairpin assembly indirectly covalent on Fe3O4@C nanoparticles with signal amplification for intracellular detection of miRNA. Talanta 223, 121675. doi: 10.1016/j.talanta.2020.121675 33303136

[B30] Feldman M. Sionov R. Smoum R. Mechoulam R. Ginsburg I. Steinberg D. (2020). Comparative Evaluation of Combinatory Interaction between Endocannabinoid System Compounds and Poly-L-lysine against Streptococcus mutans Growth and Biofilm Formation. BioMed. Res. Int. 2020, 1–7. doi: 10.1155/2020/7258380 PMC7013284 32076613

[B31] Feng T. Ai X. Ong H. Zhao Y. (2016). Dual-responsive carbon dots for tumor extracellular microenvironment triggered targeting and enhanced anticancer drug delivery. ACS Appl. Mater. Interfaces 8, 18732–18740. doi: 10.1021/acsami.6b06695 27367152

[B32] Feng H. Qian Z. (2018). Functional carbon quantum dots: A versatile platform for chemosensing and biosensing. Chem. Rec. 18, 491–505. doi: 10.1002/tcr.201700055 29171708

[B33] Fidel P. L. (2011). Candida-host interactions in HIV disease: implications for oropharyngeal candidiasis. Adv. Dent. Res. 23, 45–49. doi: 10.1177/0022034511399284 21441480 PMC3144040

[B34] Flemming H. C. Wingender J. Szewzyk U. Steinberg P. Rice S. A. Kjelleberg S. (2016). Biofilms: An emergent form of bacterial life. Nat. Rev. Microbiol. 14, 563–575. doi: 10.1038/nrmicro.2016.94 27510863

[B35] Fouad A. F. Zerella J. Barry J. Spångberg L. S. (2005). Molecular detection of Enterococcus species in root canals of therapy-resistant endodontic infections. Oral. Surg. Oral. Med. Oral. Pathol. Oral. Radiol. Endod. 99, 112–118. doi: 10.1016/j.tripleo.2004.06.064 15599358

[B36] Gawdat S. I. Bedier M. M. (2022). Influence of dual rinse irrigation on dentinal penetration of a bioceramic root canal sealer: A Conofocal microscopic Analysis. Aust. Endod. J. 48, 481–486. doi: 10.1111/aej.12599 34919319

[B37] Ghirardello M. Ramos-Soriano J. Galan M. C. (2021). Carbon dots as an emergent class of antimicrobial agents. Nanomaterials 11, 1–24. doi: 10.3390/nano11081877 PMC8400628 34443713

[B38] Gong P. Sun L. Wang F. Liu X. Yan Z. Wang M.. (2019). Highly fluorescent N-doped carbon dots with two-photon emission for ultrasensitive detection of tumor marker and visual monitor anticancer drug loading and delivery. Chem. Eng. J. 356, 994–1002. doi: 10.1016/j.cej.2018.09.100

[B39] Haffajee A. D. Socransky S. S. (1994). Microbial etiological agents of destructive periodontal diseases. Periodontol. 2000 5, 78–111. doi: 10.1111/j.1600-0757.1994.tb00020.x 9673164

[B40] Han D. Ma M. Han Y. Cui Z. Liang Y. Liu X.. (2020). Eco-friendly hybrids of carbon quantum dots modified moS2 for rapid microbial inactivation by strengthened photocatalysis. ACS Sustain. Chem. Eng. 8, 534–542. doi: 10.1021/acssuschemeng.9b06045

[B41] Havrdova M. Hola K. Skopalik J. TOmankova K. Petr M. Cepe K.. (2016). Toxicity of carbon dots-Effect of surface functionalization on the cell viability, reactive oxygen species generation and cell cycle. Carbon N. Y. 99, 238–248. doi: 10.1016/j.carbon.2015.12.027

[B42] He D. Zhang X. Yao X. Yang Y. (2022). *In vitro* and in *vivo* highly effective antibacterial activity of carbon dots-modified TiO2 nanorod arrays on titanium. Colloids Surfaces B Biointerfaces 211, 112318. doi: 10.1016/j.colsurfb.2022.112318 35007856

[B43] Hidalgo K. J. R. Carmello1 J. C. Jordão C. C. Barbugli P. A. de S. C. A. de O. E. G.. (2019). Antimicrobial photodynamic therapy in combination with nystatin in the treatment of experimental oral candidiasis induced by candida albicans resistant to fluconazole. Pharmaceuticals 12, 140. doi: 10.3390/ph12030140 31540476 PMC6789856

[B44] Högberg L. D. Heddini A. Cars O. (2010). The global need for effective antibiotics: Challenges and recent advances. Trends Pharmacol. Sci. 31, 509–515. doi: 10.1016/j.tips.2010.08.002 20843562

[B45] Hou Z. Zhang Y. Deng K. Chen Y. Li X. Deng X.. (2015). UV-emitting upconversion-based tiO2 photosensitizing nanoplatform: near-infrared light mediated in vivo photodynamic therapy via mitochondria-involved apoptosis pathway. ACS Nano 9, 2584–2599. doi: 10.1021/nn506107c 25692960

[B46] Hu C. Wang L. L. Lin Y. Q. Liang H. M. Zhou S. Y. Zheng F.. (2019). Nanoparticles for the treatment of oral biofilms: current state, mechanisms, influencing factors, and prospects. Adv. Healthc. Mater. 8, 1–23. doi: 10.1002/adhm.201901301 31763779

[B47] Hua X. W. Bao Y. W. Wang H. Y. Chen Z. Wu F. G. (2017). Bacteria-derived fluorescent carbon dots for microbial live/dead differentiation. Nanoscale 9, 2150–2161. doi: 10.1039/c6nr06558a 27874123

[B48] Huang L. Xuan Y. Koide Y. Zhiyentayev T. Tanaka M. Hamblin M. R. (2012). Type i and Type II mechanisms of antimicrobial photodynamic therapy: An in *vitro* study on gram-negative and gram-positive bacteria. Lasers Surg. Med. 44, 490–499. doi: 10.1002/lsm.22045 22760848 PMC3428129

[B49] Huang Z. Zhou T. Yuan Y. Natalie Kłodzińska S. Zheng T. Sternberg C.. (2020). Synthesis of carbon quantum dot-poly lactic-co-glycolic acid hybrid nanoparticles for chemo-photothermal therapy against bacterial biofilms. J. Colloid Interface Sci. 577, 66–74. doi: 10.1016/j.jcis.2020.05.067 32473477

[B50] Jabra-Rizk M. A. Falkler W. A. Meiller T. F. (2004). Fungal biofilms and drug resistance. Emerg. Infect. Dis. 10, 14–19. doi: 10.3201/eid1001.030119 15078591 PMC3031105

[B51] Jain P. Hassan N. Khatoon K. Mirza M. A. Naseef P. P. Kuruniyan M. S.. (2021). Periodontitis and systemic disorder—an overview of relation and novel treatment modalities. Pharmaceutics 13, 1–23. doi: 10.3390/pharmaceutics13081175 PMC8398110 34452136

[B52] Ji Y. Han Z. Ding H. Xu X. Wang D. Zhu Y.. (2021). Enhanced eradication of bacterial/fungi biofilms by glucose oxidase-modified magnetic nanoparticles as a potential treatment for persistent endodontic infections. ACS Appl. Mater. Interfaces 13, 17289–17299. doi: 10.1021/acsami.1c01748 33827209

[B53] Jia M. Yi B. Chen X. Xu Y. Xu X. Wu Z.. (2023). Carbon dots induce pathological damage to the intestine via causing intestinal flora dysbiosis and intestinal inflammation. J. Nanobiotechnol. 21, 1–17. doi: 10.1186/s12951-023-01931-1 PMC10210306 37231475

[B54] Jiang Y. Xu X. Lu J. Yin C. Li G. Bai L.. (2023). Development of ϵ-poly(L-lysine) carbon dots-modified magnetic nanoparticles and their applications as novel antibacterial agents. Front. Chem. 11. doi: 10.3389/fchem.2023.1184592 PMC10119404 37090244

[B55] Jijie R. Barras A. Bouckaert J. Dumitrascu N. Szunerits S. Boukherroub R. (2018). Enhanced antibacterial activity of carbon dots functionalized with ampicillin combined with visible light triggered photodynamic effects. Colloids Surfaces B Biointerfaces 170, 347–354. doi: 10.1016/j.colsurfb.2018.06.040 29940501

[B56] Kamada N. Chen G. Y. Inohara N. Núñez G. (2013). Control of pathogens and pathobionts by the gut microbiota. Nat. Immunol. 14, 685–690. doi: 10.1038/ni.2608 23778796 PMC4083503

[B57] Kamaruzzaman N. F. Kendall S. Good L. (2017). Targeting the hard to reach: challenges and novel strategies in the treatment of intracellular bacterial infections. Br. J. Pharmacol. 174, 2225–2236. doi: 10.1111/bph.13664 27925153 PMC5481648

[B58] Kojic E. M. Darouiche R. O. (2004). Candida infections of medical devices. Clin. Microbiol. Rev. 17, 255–267. doi: 10.1128/CMR.17.2.255-267.2004 15084500 PMC387407

[B59] Kolenbrander P. E. Palmer R. J. Periasamy S. Jakubovics N. S. (2010). Oral multispecies biofilm development and the key role of cell-cell distance. Nat. Rev. Microbiol. 8, 471–480. doi: 10.1038/nrmicro2381 20514044

[B60] Kong E. F. Kucharíková S. Van Dijck P. Peters B. M. Shirtliff M. E. Jabra-Rizka M. A. (2015). Clinical implications of oral candidiasis: Host tissue damage and disseminated bacterial disease. Infect. Immun. 83, 604–613. doi: 10.1128/IAI.02843-14 25422264 PMC4294238

[B61] Krzyściak W. Jurczak A. Kościelniak D. Bystrowska B. Skalniak A. (2014). The virulence of Streptococcus mutans and the ability to form biofilms. Eur. J. Clin. Microbiol. Infect. Dis. 33, 499–515. doi: 10.1007/s10096-013-1993-7 24154653 PMC3953549

[B62] Kumar N. Chauhan N. S. Mittal A. Sharma S. (2018). TiO 2 and its composites as promising biomaterials: a review. BioMetals 31, 147–159. doi: 10.1007/s10534-018-0078-6 29392447

[B63] Lamont R. J. Koo H. Hajishengallis G. (2018). The oral microbiota: dynamic communities and host interactions. Nat. Rev. Microbiol. 16, 745–759. doi: 10.1038/s41579-018-0089-x 30301974 PMC6278837

[B64] Larsen T. Fiehn N. E. (2017). Dental biofilm infections – an update. Apmis 125, 376–384. doi: 10.1111/apm.12688 28407420

[B65] Li L. Chen L. Lu Y. Li B. Hu R. Huang L.. (2022b). Aggregated carbon dots-loaded macrophages treat sepsis by eliminating multidrug-resistant bacteria and attenuating inflammation. Aggregate 2022, 1–15. doi: 10.1002/agt2.200

[B66] Li P. Han F. Cao W. Zhang G. Li J. Zhou J.. (2020a). Carbon quantum dots derived from lysine and arginine simultaneously scavenge bacteria and promote tissue repair. Appl. Mater. Today 19, 100601. doi: 10.1016/j.apmt.2020.100601

[B67] Li Y. J. Harroun S. G. Su Y. C. Huang C. F. Unnikrishnan B. Lin H. J.. (2016). Synthesis of self-assembled spermidine-carbon quantum dots effective against multidrug-resistant bacteria. Adv. Healthc. Mater. 5, 2545–2554. doi: 10.1002/adhm.201600297 27448287

[B68] Li H. Huang J. Song Y. Zhang M. Wang H. Lu F.. (2018a). Degradable carbon dots with broad-spectrum antibacterial activity. ACS Appl. Mater. Interfaces 10, 26936–26946. doi: 10.1021/acsami.8b08832 30039700

[B69] Li X. Huang R. Tang F. K. Li W. C. Wong S. S. W. Leung K. C. F.. (2019). Red-emissive guanylated polyene-functionalized carbon dots arm oral epithelia against invasive fungal infections. ACS Appl. Mater. Interfaces 11, 46591–46603. doi: 10.1021/acsami.9b18003 31742377

[B70] Li Y. Liu W. Sun C. Zheng M. Zhang J. Liu B.. (2018b). Hybrids of carbon dots with subunit B of ricin toxin for enhanced immunomodulatory activity. J. Colloid Interface Sci. 523, 226–233. doi: 10.1016/j.jcis.2018.03.108 29626760

[B71] Li H. Wei W. Xu H. (2022a). Drug discovery is an eternal challenge for the biomedical sciences. Acta Mater. Med. 1, 1–3. doi: 10.15212/amm-2022-1001

[B72] Li X. Wu X. Yuan T. Zhu J. Yang Y. (2021b). Influence of the iodine content of nitrogen- and iodine-doped carbon dots as a peroxidase mimetic nanozyme exhibiting antifungal activity against C. albicans. Biochem. Eng. J. 175, 108139. doi: 10.1016/j.bej.2021.108139

[B73] Li P. Yang X. Zhang X. Pan J. Tang W. Cao W.. (2020b). Surface chemistry-dependent antibacterial and antibiofilm activities of polyamine-functionalized carbon quantum dots. J. Mater. Sci. 55, 16744–16757. doi: 10.1007/s10853-020-05262-6

[B74] Li N. Zhang J. Wang C. Sun H. (2017). Enhanced photocatalytic degradation of tetrabromobisphenol A by tourMaline–TiO2 composite catalyst. J. Mater. Sci. 52, 6937–6949. doi: 10.1007/s10853-017-0926-8

[B75] Li B. Zhao S. Huang L. Wang Q. Xiao J. Lan M. (2021a). Recent advances and prospects of carbon dots in phototherapy. Chem. Eng. J. 408, 127245. doi: 10.1016/j.cej.2020.127245

[B76] Liang J. Peng X. Zhou X. Zou J. Cheng L. (2020b). Emerging applications of drug delivery systems in oral infectious diseases prevention and treatment. Molecules 25, 516. doi: 10.3390/molecules25030516 31991678 PMC7038021

[B77] Liang G. Shi H. Qi Y. Li J. Jing A. Liu Q.. (2020a). Specific anti-biofilm activity of carbon quantum dots by destroying p. Gingivalis biofilm related genes. Int. J. Nanomed. 15, 5473–5489. doi: 10.2147/IJN.S253416 PMC7406331 32801701

[B78] Liguori G. R. Zhou Q. Liguori T. T. A. Barros G. G. Kühn P. T. Moreira L. F. P.. (2019). Directional topography influences adipose mesenchymal stromal cell plasticity: Prospects for tissue engineering and fibrosis. Stem Cells Int. 2019, 5387850. doi: 10.1155/2019/5387850 31191675 PMC6525798

[B79] Lin F. Bao Y.-W. Wu F.-G. (2019). Carbon dots for sensing and killing microorganisms. J. Carbon Res. 5, 33. doi: 10.3390/c5020033

[B80] Lin F. Li C. Dong L. Fu D. Chen Z. (2017). Imaging biofilm-encased microorganisms using carbon dots derived from L. plantarum. Nanoscale 9, 9056–9064. doi: 10.1039/c7nr01975k 28639672

[B81] Listgarten M. A. (1986). Pathogenesis of periodontitis. J. Clin. Periodontol. 13, 418–425. doi: 10.1111/j.1600-051X.1986.tb01485.x 3522650

[B82] Liu M. Chen M. Yang Z. (2017b). Design of amphotericin B oral formulation for antifungal therapy. Drug Deliv. 24, 1–9. doi: 10.1080/10717544.2016.1225852 PMC8241147 28155335

[B83] Liu L. Han Z. An F. Gong X. Zhao C. Zheng W.. (2021a). Aptamer-based biosensors for the diagnosis of sepsis. J. Nanobiotechnol. 19, 1–22. doi: 10.1186/s12951-021-00959-5 PMC8287673 34281552

[B84] Liu M. Huang L. Xu X. Wei X. Yang X. Li X.. (2022). Copper doped carbon dots for addressing bacterial biofilm formation, wound infection, and tooth staining. ACS Nano 16, 9479–9497. doi: 10.1021/acsnano.2c02518 35713471

[B85] Liu J. Lu S. Tang Q. Zhang K. Yu W. Sun H.. (2017a). One-step hydrothermal synthesis of photoluminescent carbon nanodots with selective antibacterial activity against Porphyromonas gingivalis. Nanoscale 9, 7135–7142. doi: 10.1039/c7nr02128c 28513713

[B86] Liu S. Quan T. Yang L. Deng L. Kang X. Gao M.. (2021b). N,Cl-codoped carbon dots from impatiens balsamina L. Stems and a deep eutectic solvent and their applications for gram-positive bacteria identification, antibacterial activity, cell imaging, and clO-sensing. ACS Omega 6, 29022–29036. doi: 10.1021/acsomega.1c04078 34746591 PMC8567351

[B87] Liu Y. Y. Yu N. Y. Fang W. Tan Q. G. Ji R. Yang L. Y.. (2021c). Photodegradation of carbon dots cause cytotoxicity. Nat. Commun. 12, 1–12. doi: 10.1038/s41467-021-21080-z 33547279 PMC7864953

[B88] Löfmark S. Edlund C. Nord C. E. (2010). Metronidazole is still the drug of choice for treatment of anaerobic infections. Clin. Infect. Dis. 50, S16–S23. doi: 10.1086/647939 20067388

[B89] Lu F. Ma Y. Huang H. Zhang Y. Kong H. Zhao Y.. (2021). Edible and highly biocompatible nanodots from natural plants for the treatment of stress gastric ulcers. Nanoscale 13, 6809–6818. doi: 10.1039/d1nr01099a 33885482

[B90] Mai B. Wang X. Liu Q. Leung A. W. Wang X. Xu C.. (2016). The antibacterial effect of sinoporphyrin sodium photodynamic therapy on Staphylococcus aureus planktonic and biofilm cultures. Lasers Surg. Med. 48, 400–408. doi: 10.1002/lsm.22468 26749227

[B91] Makabenta J. M. V. Nabawy A. Li C. H. Schmidt-Malan S. Patel R. Rotello V. M. (2021). Nanomaterial-based therapeutics for antibiotic-resistant bacterial infections. Nat. Rev. Microbiol. 19, 23–36. doi: 10.1038/s41579-020-0420-1 32814862 PMC8559572

[B92] Makkawi H. Hoch S. Burns E. Hosur K. Hajishengallis G. Kirschning C. J.. (2017). Porphyromonas gingivalis stimulates TLR2-PI3K signaling to escape immune clearance and induce bone resorption independently of MyD88. Front. Cell. Infect. Microbiol. 7. doi: 10.3389/fcimb.2017.00359 PMC5550410 28848717

[B93] Matsuzaki K. (2009). Control of cell selectivity of antimicrobial peptides. Biochim. Biophys. Acta - Biomembr. 1788, 1687–1692. doi: 10.1016/j.bbamem.2008.09.013 18952049

[B94] Mei L. Zhang D. Shao H. Hao Y. Zhang T. Zheng W.. (2022). Injectable and self-healing probiotics-loaded hydrogel for promoting superbacteria-infected wound healing. ACS Appl. Mater. Interfaces 14, 20538–20550. doi: 10.1021/acsami.1c23713 35471815

[B95] Meisel P. Kocher T. (2005). Photodynamic therapy for periodontal diseases: State of the art. J. Photochem. Photobiol. B Biol. 79, 159–170. doi: 10.1016/j.jphotobiol.2004.11.023 15878121

[B96] Melo M. A. S. Rolim J. P. M. L. Passos V. F. Lima R. A. Zanin I. C. J. Codes B. M.. (2015). Photodynamic antimicrobial chemotherapy and ultraconservative caries removal linked for management of deep caries lesions. Photodiagnosis Photodyn. Ther. 12, 581–586. doi: 10.1016/j.pdpdt.2015.09.005 26431977

[B97] Mima E. G. Vergani C. E. MaChado A. L. Massucato E. M. S. Colombo A. L. Bagnato V. S.. (2012). Comparison of Photodynamic Therapy versus conventional antifungal therapy for the treatment of denture stomatitis: A randomized clinical trial. Clin. Microbiol. Infect. 18, E380–E388. doi: 10.1111/j.1469-0691.2012.03933.x 22731617

[B98] Miramoth N. S. Di Meo C. Zouhiri F. Saïd-Hassane F. Valetti S. Gorges R.. (2012). Self-assembled squalenoylated penicillin bioconjugates: An original approach for the treatment of intracellular infections. ACS Nano 6, 3820–3831. doi: 10.1021/nn204928v 22482704

[B99] Montes L. F. Wilborn W. H. (1968). Ultrastructural features of host-parasite relationship in oral candidiasis. J. Bacteriol. 96, 1349–1356. doi: 10.1128/jb.96.4.1349-1356.1968 5686004 PMC252453

[B100] Moradlou O. Rabiei Z. Delavari N. (2019). Antibacterial effects of carbon quantum dots@hematite nanostructures deposited on titanium against Gram-positive and Gram-negative bacteria. J. Photochem. Photobiol. A Chem. 379, 144–149. doi: 10.1016/j.jphotochem.2019.04.047

[B101] Naglik J. R. Tang S. X. Moyes D. L. (2013). Oral colonization of fungi. Curr. Fungal Infect. Rep. 7, 152–159. doi: 10.1007/s12281-013-0129-y

[B102] Nie X. Jiang C. Wu S. Chen W. Lv P. Wang Q.. (2020). Carbon quantum dots: A bright future as photosensitizers for in *vitro* antibacterial photodynamic inactivation. J. Photochem. Photobiol. B Biol. 206, 111864. doi: 10.1016/j.jphotobiol.2020.111864 32247250

[B103] O’Brien-Simpson N. M. Veith P. D. Dashper S. G. Reynolds E. C. (2004). Antigens of bacteria associated with periodontitis. Periodontol. 2000 35, 101–134. doi: 10.1111/j.0906-6713.2004.003559.x 15107060

[B104] Ostadhossein F. Moitra P. Altun E. Dutta D. Sar D. Tripathi I.. (2021). Function-adaptive clustered nanoparticles reverse Streptococcus mutans dental biofilm and maintain microbiota balance. Commun. Biol. 4, 1–16. doi: 10.1038/s42003-021-02372-y 34267305 PMC8282845

[B105] Pan J. Sheng Y. Zhang J. Wei J. Huang P. Zhang X.. (2014). Preparation of carbon quantum dots/ TiO2 nanotubes composites and their visible light catalytic applications. J. Mater. Chem. A 2, 18082–18086. doi: 10.1039/c4ta03528c

[B106] Pang W. Jiang P. Ding S. Bao Z. Wang N. Wang H.. (2020). Nucleolus-targeted photodynamic anticancer therapy using renal-clearable carbon dots. Adv. Healthc. Mater. 9, 1–8. doi: 10.1002/adhm.202000607 32548916

[B107] Pantlin L. (2008). Is there a role for antibiotics in periodontal treatment? Dent. Update 35, 493–496. doi: 10.12968/denu.2008.35.7.493 18853720

[B108] Parhizkar A. Nojehdehian H. Asgary S. (2018). Triple antibiotic paste: momentous roles and applications in endodontics: a review. Restor. Dent. Endod. 43, 1–16. doi: 10.5395/rde.2018.43.e28 PMC6103545 30135847

[B109] Patil S. Rao R. S. Majumdar B. Anil S. (2015). Clinical appearance of oral Candida infection and therapeutic strategies. Front. Microbiol. 6. doi: 10.3389/fmicb.2015.01391 PMC4681845 26733948

[B110] Persoon I. F. Buijs M. J. Özok A. R. Crielaard W. Krom B. P. Zaura E.. (2017). The mycobiome of root canal infections is correlated to the bacteriome. Clin. Oral. Investig. 21, 1871–1881. doi: 10.1007/s00784-016-1980-3 PMC5442261 27771826

[B111] Persson G. R. Renvert S. (2014). Cluster of bacteria associated with peri-implantitis. Clin. Implant Dent. Relat. Res. 16, 783–793. doi: 10.1111/cid.12052 23527870

[B112] Peulen T. O. Wilkinson K. J. (2011). Diffusion of nanoparticles in a biofilm. Environ. Sci. Technol. 45, 3367–3373. doi: 10.1021/es103450g 21434601

[B113] Pinďáková L. Kašpárková V. Kejlová K. Dvořáková M. Krsek D. Jírová D.. (2017). Behaviour of silver nanoparticles in simulated saliva and gastrointestinal fluids. Int. J. Pharm. 527, 12–20. doi: 10.1016/j.ijpharm.2017.05.026 28506800

[B114] Pokrowiecki R. Wojnarowicz J. Zareba T. Koltsov I. Lojkowski W. Tyski S.. (2019). Nanoparticles and human saliva: A step towards drug delivery systems for dental and craniofacial biomaterials. Int. J. Nanomed. 14, 9235–9257. doi: 10.2147/IJN.S221608 PMC6886554 31819427

[B115] Popova C. Dosseva-Panova V. Panov V. (2013). Microbiology of periodontal diseases. A review. Biotechnol. Biotechnol. Equip. 27, 3754–3759. doi: 10.5504/BBEQ.2013.0027

[B116] Pourhajibagher M. Parker S. Chiniforush N. Bahador A. (2019). Photoexcitation triggering via semiconductor Graphene Quantum Dots by photochemical doping with Curcumin versus perio-pathogens mixed biofilms. Photodiagnosis Photodyn. Ther. 28, 125–131. doi: 10.1016/j.pdpdt.2019.08.025 31479805

[B117] Preshaw P. M. Seymour R. A. Heasman P. A. (2005). Current concepts in periodontal pathogenesis. Periodontology 31, 570–578. doi: 10.12968/denu.2004.31.10.570 15656071

[B118] Priyadarshini E. Rawat K. Prasad T. Bohidar H. B. (2018). Antifungal efficacy of Au@ carbon dots nanoconjugates against opportunistic fungal pathogen, Candida albicans. Colloids Surfaces B Biointerfaces 163, 355–361. doi: 10.1016/j.colsurfb.2018.01.006 29335197

[B119] Raina S. Thakur A. Sharma A. Pooja D. Minhas A. P. (2020). Bactericidal activity of Cannabis sativa phytochemicals from leaf extract and their derived Carbon Dots and Ag@Carbon Dots. Mater. Lett. 262, 127122. doi: 10.1016/j.matlet.2019.127122

[B120] Ramage G. Walle K. V. Wickes B. L. López-Ribot J. L. (2001). Biofilm formation by Candida dubliniensis. J. Clin. Microbiol. 39, 3234–3240. doi: 10.1128/JCM.39.9.3234-3240.2001 11526156 PMC88324

[B121] Ran H. H. Cheng X. Bao Y. W. Hua X. W. Gao G. Zhang X.. (2019). Multifunctional quaternized carbon dots with enhanced biofilm penetration and eradication efficiencies. J. Mater. Chem. B 7, 5104–5114. doi: 10.1039/c9tb00681h 31432881

[B122] Rao B. C. Zhang G. Z. Zou Y. W. Ren T. Ren H. Y. Liu C.. (2022). Alterations in the human oral microbiome in cholangiocarcinoma. Mil. Med. Res. 9, 22–25. doi: 10.1186/s40779-022-00423-x 36345047 PMC9641929

[B123] Ré A. C. S. Martins J. F. Cunha-Filho M. Gelfuso G. M. Aires C. P. Gratieri T. (2021). New perspectives on the topical management of recurrent candidiasis. Drug Deliv. Transl. Res. 11, 1568–1585. doi: 10.1007/s13346-021-00901-0 33469892

[B124] Rickard A. H. Gilbert P. High N. J. Kolenbrander P. E. Handley P. S. (2003). Bacterial coaggregation: An integral process in the development of multi-species biofilms. Trends Microbiol. 11, 94–100. doi: 10.1016/S0966-842X(02)00034-3 12598132

[B125] Ritenberg M. Nandi S. Kolusheva S. Dandela R. Meijler M. M. Jelinek R. (2016). Imaging pseudomonas aeruginosa biofilm extracellular polymer scaffolds with amphiphilic carbon dots. ACS Chem. Biol. 11, 1265–1270. doi: 10.1021/acschembio.5b01000 26882175

[B126] Romero M. P. Alves F. Stringasci M. D. Buzzá H. H. Ciol H. Inada N. M.. (2021). One-Pot Microwave-Assisted Synthesis of Carbon Dots and in *vivo* and in *vitro* Antimicrobial Photodynamic Applications. Front. Microbiol. 12. doi: 10.3389/fmicb.2021.662149 PMC8255795 34234756

[B127] Sah A. K. Dewangan M. Suresh P. K. (2019). Potential of chitosan-based carrier for periodontal drug delivery. Colloids Surfaces B Biointerfaces 178, 185–198. doi: 10.1016/j.colsurfb.2019.02.044 30856588

[B128] Samaranayake L. Matsubara V. H. (2017). Normal oral flora and the oral ecosystem. Dent. Clin. North Am. 61, 199–215. doi: 10.1016/j.cden.2016.11.002 28317562

[B129] Sattarahmady N. Rezaie-Yazdi M. Tondro G. H. Akbari N. (2017). Bactericidal laser ablation of carbon dots: An in *vitro* study on wild-type and antibiotic-resistant Staphylococcus aureus. J. Photochem. Photobiol. B Biol. 166, 323–332. doi: 10.1016/j.jphotobiol.2016.12.006 28024283

[B130] Scheibler E. Garcia M. C. R. Medina da Silva R. Figueiredo M. A. Salum F. G. Cherubini K. (2017). Use of nystatin and chlorhexidine in oral medicine: Properties, indications and pitfalls with focus on geriatric patients. Gerodontology 34, 291–298. doi: 10.1111/ger.12278 28556195

[B131] Scialabba C. Sciortino A. Messina F. Buscarino G. Cannas M. Roscigno G.. (2019). Highly homogeneous biotinylated carbon nanodots: red-emitting nanoheaters as theranostic agents toward precision cancer medicine. ACS Appl. Mater. Interfaces 11, 19854–19866. doi: 10.1021/acsami.9b04925 31088077

[B132] Shaikh A. F. Tamboli M. S. Patil R. H. Bhan A. Ambekar J. D. Kale B. B. (2018). Bioinspired carbon quantum dots: an antibiofilm agents. J. Nanosci. Nanotechnol. 19, 2339–2345. doi: 10.1166/jnn.2019.16537 30486995

[B133] Shao Q. Ding T. Pan F. Li G. Shen S. Qian J.. (2022). Protein corona mediated liposomal drug delivery for bacterial infection management. Asian J. Pharm. Sci. 17, 855–866. doi: 10.1016/j.ajps.2022.10.003 36600900 PMC9800951

[B134] Sheng G. Tian N. Duan H. Sun Z. Chu H. (2022). Advances in therapeutic nanodrug delivery systems for infectious lung diseases: a review. Acta Mater. Med. 1, 343–364. doi: 10.15212/amm-2022-0019

[B135] Sidhu J. S. Mayank Pandiyan T. Kaur N. Singh N. (2017). The photochemical degradation of bacterial cell wall using penicillin-based carbon dots: weapons against multi-drug resistant (MDR) strains. ChemistrySelect 2, 9277–9283. doi: 10.1002/slct.201701810

[B136] Siqueira J. F. De Uzeda M. (1996). Disinfection by calcium hydroxide pastes of dentinal tubules infected with two obligate and one facultative anaerobic bacteria. J. Endod. 22, 674–676. doi: 10.1016/S0099-2399(96)80062-8 9220753

[B137] Siqueira J. F. Lopes H. P. (1999). Mechanisms of antimicrobial activity of calcium hydroxide: A critical review. Int. Endod. J. 32, 361–369. doi: 10.1046/j.1365-2591.1999.00275.x 10551109

[B138] Slots J. Rams T. E. (1990). Antibiotics in periodontal therapy: advantages and disadvantages. J. Clin. Periodontol. 17, 479–493. doi: 10.1111/j.1365-2710.1992.tb01220.x 2202744

[B139] Smith A. J. Jackson M. S. Bagg J. (2001). The ecology of staphylococcus species in the oral cavity. J. Med. Microbiol. 50, 940–946. doi: 10.1099/0022-1317-50-11-940 11699589

[B140] Soltani R. Guo S. Bianco A. Ménard-Moyon C. (2020). Carbon nanomaterials applied for the treatment of inflammatory diseases: preclinical evidence. Adv. Ther. 3, 1–26. doi: 10.1002/adtp.202000051

[B141] Song W. Jia P. Zhang T. Dou K. Liu L. Ren Y.. (2022). Cell membrane-camouflaged inorganic nanoparticles for cancer therapy. J. Nanobiotechnol. 20, 1–29. doi: 10.1186/s12951-022-01475-w PMC9206402 35717234

[B142] Stokes J. M. Lopatkin A. J. Lobritz M. A. Collins J. J. (2019). Bacterial metabolism and antibiotic efficacy. Cell Metab. 30, 251–259. doi: 10.1016/j.cmet.2019.06.009 31279676 PMC6990394

[B143] Su W. Wu H. Xu H. Zhang Y. Li Y. Li X.. (2020). Carbon dots: A booming material for biomedical applications. Mater. Chem. Front. 4, 821–836. doi: 10.1039/c9qm00658c

[B144] Sun B. Wu F. Zhang Q. Chu X. Wang Z. Huang X.. (2021). Insight into the effect of particle size distribution differences on the antibacterial activity of carbon dots. J. Colloid Interface Sci. 584, 505–519. doi: 10.1016/j.jcis.2020.10.015 33129160

[B145] Sundaram D. Narayanan R. K. Vadakkepurayil K. (2016). A comparative evaluation on antimicrobial effect of honey, neem leaf extract and sodium hypochlorite as intracanal irrigant: An ex-vivo study. J. Clin. Diagn. Res. 10, ZC88–ZC91. doi: 10.7860/JCDR/2016/19268.8311 PMC5028579 27656571

[B146] Swidergall M. Filler S. G. (2017). Oropharyngeal candidiasis: fungal invasion and epithelial cell responses. PloS Pathog. 13, 1–7. doi: 10.1371/journal.ppat.1006056 PMC5230744 28081240

[B147] Takeuchi H. Furuta N. Morisaki I. Amano A. (2011). Exit of intracellular Porphyromonas gingivalis from gingival epithelial cells is mediated by endocytic recycling pathway. Cell. Microbiol. 13, 677–691. doi: 10.1111/j.1462-5822.2010.01564.x 21155963

[B148] Talianu M. Ghica M. V. Anut V. (2022). Molecular mapping of antifungal mechanisms accessing biomaterials and new agents to target oral candidiasis. Int. J. Mol. Sci. 23, 7520. doi: 10.3390/ijms23147520 35886869 PMC9320712

[B149] Tang S. Zhang H. Mei L. Dou K. Jiang Y. Sun Z.. (2022). Fucoidan − derived carbon dots against Enterococcus faecalis biofilm and infected dentinal tubules for the treatment of persistent endodontic infections. J. Nanobiotechnol. 20, 321. doi: 10.1186/s12951-022-01501-x PMC9281061 35836267

[B150] Taufiq H. R. Mirza M. Paramita S. A. Sutanto H. (2020). Synthesis of ZnO/C dots as antibacterial dental bracket. IOP Conf. Ser. Mater. Sci. Eng. 850, 12045. doi: 10.1088/1757-899X/850/1/012045

[B151] Tennert C. Feldmann K. Haamann E. Al-Ahmad A. Follo M. Wrbas K. T.. (2014). Effect of photodynamic therapy (PDT) on Enterococcus faecalis biofilm in experimental primary and secondary endodontic infections. BMC Oral. Health 14, 1–8. doi: 10.1186/1472-6831-14-132 25366394 PMC4236465

[B152] Teymourinia H. Amiri O. Salavati-Niasari M. (2021). Synthesis and characterization of cotton-silver-graphene quantum dots (cotton/Ag/GQDs) nanocomposite as a new antibacterial nanopad. Chemosphere 267, 129293. doi: 10.1016/j.chemosphere.2020.129293 33348263

[B153] Thakur M. Pandey S. Mewada A. Patil V. Khade M. Goshi E.. (2014). Antibiotic conjugated fluorescent carbon dots as a theranostic agent for controlled drug release, bioimaging, and enhanced antimicrobial activity. J. Drug Deliv. 2014, 1–9. doi: 10.1155/2014/282193 PMC3976943 24744921

[B154] Tosco V. Monterubbianesi R. Aranguren J. Memè L. Putignano A. Orsini G. (2023). Evaluation of the efficacy of different irrigation systems on the removal of root canal smear layer: A scanning electron microscopic study. Appl. Sci. 13, 149. doi: 10.3390/app13010149

[B155] Travlou N. A. Giannakoudakis D. A. Algarra M. Labella A. M. Rodríguez-Castellón E. Bandosz T. J. (2018). S- and N-doped carbon quantum dots: Surface chemistry dependent antibacterial activity. Carbon N. Y. 135, 104–111. doi: 10.1016/j.carbon.2018.04.018 34996200

[B156] Varghese M. Balachandran M. (2021). Antibacterial efficiency of carbon dots against Gram-positive and Gram-negative bacteria: A review. J. Environ. Chem. Eng. 9, 106821. doi: 10.1016/j.jece.2021.106821

[B157] Veiga N. J. Aires D. Douglas F. Pereira M. Vaz A. Rama L.. (2016). Dental caries: a review. J. Dent. Oral. Heal. 2, 2–4. doi: 10.15272/ajbps.v6i53.773

[B158] Wang C. Xie J. Dong X. Mei L. Zhao M. Leng Z.. (2020). Clinically approved carbon nanoparticles with oral administration for intestinal radioprotection via protecting the small intestinal crypt stem cells and maintaining the balance of intestinal flora. Small 16, 1906915. doi: 10.1002/smll.201906915 32187855

[B159] Wayakanon K. Thornhill M. H. Douglas C. W. I. Lewis A. L. Warren N. J. Pinnock A.. (2013). Polymersome-mediated intracellular delivery of antibiotics to treat Porphyromonas gingivalis-infected oral epithelial cells. FASEB J. 27, 4455–4465. doi: 10.1096/fj.12-225219 23921377

[B160] Wei X. Cheng F. Yao Y. Yi X. Wei B. Li H.. (2021). Facile synthesis of a carbon dots and silver nanoparticles (CDs/AgNPs) composite for antibacterial application. RSC Adv. 11, 18417–18422. doi: 10.1039/d1ra02600c 35480903 PMC9033427

[B161] Wei G. Yang G. Wang Y. Jiang H. Fu Y. Yue G.. (2020). Phototherapy-based combination strategies for bacterial infection treatment. Theranostics 10, 12241–12262. doi: 10.7150/thno.52729 33204340 PMC7667673

[B162] Whitmore S. E. Lamont R. J. (2014). Oral bacteria and cancer. PloS Pathog. 10, 1–3. doi: 10.1371/journal.ppat.1003933 PMC3968118 24676390

[B163] Wu P. Liu X. Duan Y. Pan L. Sun Z. Chu H.. (2023). ZnPc photosensitizer-loaded peony-shaped FeSe2 remotely controlled by near-infrared light for antimycobacterial therapy. Acta Mater. Med. 2, 260–269. doi: 10.15212/amm-2023-0012

[B164] Xia M. Y. Xie Y. Yu C. H. Chen G. Y. Li Y. H. Zhang T.. (2019). Graphene-based nanomaterials: the promising active agents for antibiotics-independent antibacterial applications. J. Control. Release 307, 16–31. doi: 10.1016/j.jconrel.2019.06.011 31185232

[B165] Xiong M. Bao Y. Yang X. Wang Y. Sun B. Wang J. (2012). Lipase-sensitive polymeric triple-layered nanogel for “ On-demand “ Drug delivery. J. Am. Chem. Soc. 134, 4355–4362. doi: 10.1021/ja211279u 22304702

[B166] Xu Z. Liu X. W. Ma Y. S. Gao H. W. (2010). Interaction of nano-TiO2 with lysozyme: Insights into the enzyme toxicity of nanosized particles. Environ. Sci. Pollut. Res. 17, 798–806. doi: 10.1007/s11356-009-0153-1 19390888

[B167] Xu X. Ray R. Gu Y. Ploehn H. J. Gearheart L. Raker K.. (2004). Electrophoretic analysis and purification of fluorescent single-walled carbon nanotube fragments. J. Am. Chem. Soc. 126, 12736–12737. doi: 10.1021/ja040082h 15469243

[B168] Xu H. V. Zheng X. T. Wang C. Zhao Y. Tan Y. N. (2018). Bioinspired antimicrobial nanodots with amphiphilic and zwitterionic-like characteristics for combating multidrug-resistant bacteria and biofilm removal. ACS Appl. Nano Mater. 1, 2062–2068. doi: 10.1021/acsanm.8b00465

[B169] Yamatake K. Maeda M. Kadowaki T. Takii R. Tsukuba T. Ueno T.. (2007). Role for gingipains in Porphyromonas gingivalis traffic to phagolysosomes and survival in human aortic endothelial cells. Infect. Immun. 75, 2090–2100. doi: 10.1128/IAI.01013-06 17296756 PMC1865784

[B170] Yan M. Pan Y. Lu S. Li X. Wang D. Shao T.. (2022). Chitosan-CaP microflowers and metronidazole loaded calcium alginate sponges with enhanced antibacterial , hemostatic and osteogenic properties for the prevention of dry socket after tooth removal. Int. J. Biol. Macromol. 212, 134–145. doi: 10.1016/j.ijbiomac.2022.05.094 35588978

[B171] Yang J. Gao G. Zhang X. Ma Y. H. Chen X. Wu F. G. (2019). One-step synthesized carbon dots with bacterial contact-enhanced fluorescence emission property: Fast Gram-type identification and selective Gram-positive bacterial inactivation. Carbon N. Y. 146, 827–839. doi: 10.1016/j.carbon.2019.02.040

[B172] Yang X. Li P. Tang W. Du S. Yu M. Lu H.. (2021). A facile injectable carbon dot/oxidative polysaccharide hydrogel with potent self-healing and high antibacterial activity. Carbohydr. Polym. 251, 117040. doi: 10.1016/j.carbpol.2020.117040 33142598

[B173] Yang Z. Liang X. Jiang X. Guo J. Tao Y. Wang S.. (2018). Development and evaluation of minocycline hydrochloride-loaded in *situ* cubic liquid crystal for intra-periodontal pocket administration. Molecules 23, 2275. doi: 10.3390/molecules23092275 30200615 PMC6225298

[B174] Yavuz E. Dinc S. Kara M. (2020). Effects of endogenous molasses carbon dots on macrophages and their potential utilization as anti-inflammatory agents. Appl. Phys. A Mater. Sci. Process. 126, 1–10. doi: 10.1007/s00339-019-3189-1

[B175] Ye P. Chang J. Foo L. F. Yap B. C. M. (2017). An early report: A modified porphyrin-linked metronidazole targeting intracellular Porphyromonas gingivalis in cultured oral epithelial cells. Int. J. Oral. Sci. 9, 167–173. doi: 10.1038/ijos.2017.31 28960193 PMC5709547

[B176] Yılmaz S. Calikoglu E. O. Kosan Z. (2019). for an uncommon neurosurgical emergency in a developing country. Niger. J. Clin. Pract. 22, 1070–1077. doi: 10.4103/njcp.njcp 31417049

[B177] Zhang J. An X. Li X. Liao X. Nie Y. Fan Z. (2018). Enhanced antibacterial properties of the bracket under natural light via decoration with ZnO/carbon quantum dots composite coating. Chem. Phys. Lett. 706, 702–707. doi: 10.1016/j.cplett.2018.06.029

[B178] Zhang X. Pan J. Zhu C. Sheng Y. Yan Z. Wang Y.. (2015). The visible light catalytic properties of carbon quantum dots/ZnO nanoflowers composites. J. Mater. Sci. Mater. Electron. 26, 2861–2866. doi: 10.1007/s10854-015-2769-x

[B179] Zhao Y. Huang T. Zhang X. Cui Y. Zhang L. Li L.. (2023). Piezotronic and piezo-phototronic effects on sonodynamic disease therapy. BMEMat 1, e12006. doi: 10.1002/bmm2.12006

[B180] Zhao Y. Pu R. Qian Y. Shi J. Si M. (2021). Antimicrobial photodynamic therapy versus antibiotics as an adjunct in the treatment of periodontitis and peri-implantitis: A systematic review and meta-analysis. Photodiagnosis Photodyn. Ther. 34, 102231. doi: 10.1016/j.pdpdt.2021.102231 33621702

[B181] Zhao X. Tang Q. Zhu S. Bu W. Yang M. Liu X.. (2019). Controllable acidophilic dual-emission fluorescent carbonized polymer dots for selective imaging of bacteria. Nanoscale 11, 9526–9532. doi: 10.1039/c9nr01118h 31049503

[B182] Zhao W. B. Wang R. T. Liu K. K. Du M. R. Wang Y. Wang Y. Q.. (2022c). Near-infrared carbon nanodots for effective identification and inactivation of Gram-positive bacteria. Nano Res. 15, 1699–1708. doi: 10.1007/s12274-021-3818-9

[B183] Zhao C. Wang X. Yu L. Wu L. Hao X. Liu Q.. (2022a). Quaternized carbon quantum dots with broad-spectrum antibacterial activity for the treatment of wounds infected with mixed bacteria. Acta Biomater. 138, 528–544. doi: 10.1016/j.actbio.2021.11.010 34775123

[B184] Zhao D. Zhang R. Liu X. Li X. Xu M. Huang X.. (2022b). Screening of chitosan derivatives-carbon dots based on antibacterial activity and application in anti-staphylococcus aureus biofilm. Int. J. Nanomed. 17, 937–952. doi: 10.2147/IJN.S350739 PMC8904944 35280335

[B185] Zhou Q. Ge L. Guimarães C. F. Kühn P. T. Yang L. van Rijn P. (2018a). Development of a novel orthogonal double gradient for high-throughput screening of mesenchymal stem cells–materials interaction. Adv. Mater. Interfaces 5, 1–8. doi: 10.1002/admi.201800504

[B186] Zhou Q. Wünnemann P. Kühn P. T. de Vries J. Helmin M. Böker A.. (2016). Mechanical properties of aligned nanotopologies for directing cellular behavior. Adv. Mater. Interfaces 3, 1–10. doi: 10.1002/admi.201600275

[B187] Zhou Q. Zhao Z. Zhou Z. Zhang G. Chiechi R. C. van Rijn P. (2018b). Directing mesenchymal stem cells with gold nanowire arrays. Adv. Mater. Interfaces 5, 1–8. doi: 10.1002/admi.201800334

[B188] Zhu S. Song Y. Zhao X. Shao J. Zhang J. Yang B. (2015). The photoluminescence mechanism in carbon dots (graphene quantum dots, carbon nanodots, and polymer dots): current state and future perspective. Nano Res. 8, 355–381. doi: 10.1007/s12274-014-0644-3

[B189] Zhu Y. Wang Z. Bai L. Deng J. Zhou Q. (2021). Biomaterial-based encapsulated probiotics for biomedical applications: Current status and future perspectives. Mater. Des. 210, 110018. doi: 10.1016/j.matdes.2021.110018

